# Multi-Scale Traditional and Non-Traditional Machining of Bulk Metallic Glasses (BMGs)—Review of Challenges, Recent Advances, and Future Directions

**DOI:** 10.3390/mi15060686

**Published:** 2024-05-23

**Authors:** Muhammad P. Jahan, Aakash Niraula, Muhammad Abdun Nafi, Asma Perveen

**Affiliations:** 1 Department of Mechanical and Manufacturing Engineering, Miami University, Oxford, OH 45056, USA; niraula@miamioh.edu (A.N.); nafim@miamioh.edu (M.A.N.); 2 Department of Mechanical & Aerospace Engineering, Nazarbayev University, Nur-Sultan 010000, Kazakhstan; asma.perveen@nu.edu.kz

**Keywords:** bulk metallic glass (BMG), conventional machining, non-conventional machining, crystallization, current challenges, future research direction

## Abstract

Bulk metallic glasses (BMGs) are growing in popularity prominently due to their potential in micro-electromechanical systems (MEMSs) and aerospace applications. BMGs have unique mechanical properties, i.e., high strength, hardness, modulus of elasticity, and wear resistance, due to their disordered atomic structure. Due to their unique mechanical properties and amorphous structures, machining of BMGs remains a challenge. This paper aims to carry out a detailed literature review on various aspects of the machining of bulk metallic glasses using both conventional and non-conventional processes, including experimental approaches, modeling, statistical findings, challenges, and guidelines for machining this difficult-to-machine material. Conventional machining processes were found to be challenging for machining bulk metallic glasses due to their high hardness, brittleness, and tendency to convert their amorphous structure into a crystalline structure, especially at the machined surface and sub-surface. Although their high electrical conductivity makes them suitable for machining by non-conventional processes, they impose new challenges such as heat-affected zones and crystallization. Therefore, the successful machining of BMGs requires more in-depth analysis of cutting forces, tool wear, burr formation, surface finish, recast layers or heat-affected zones, crystallization, and mechanical property changes among different varieties of BMGs. This review paper provides guidelines emerging from in-depth analysis of previous studies, as well as offering directions for future research in the machining of BMGs.

## 1. Introduction

Bulk Metallic Glasses (BMGs) are metallic materials with an amorphous microstructure, which are generally produced by rapidly cooling liquid metals and alloys. Their mechanical and structural properties make metallic glasses unique in nature. Some of these properties include high hardness, high resistance to corrosion and tear, high tensile strength, high fracture toughness, low thermal and electrical conductivity, high rupture strength, and large elastic strain limit. Due to the potential of metallic glass, its applications are currently in optics, aerospace, sports, and biomedical fields.

Bulk metallic glasses are produced in mold and have the capability of forming into near net shapes. In addition, the requirement of a high cooling rate to form the glassy structure limits the structures that can be produced by the BMGs [1]. Moreover, multi-scale fabrication of complex 3D structures can be sometime challenging, due to the need for complex-shaped die design and the time associated with the machining of die. In addition, for limited production of parts and components, fabrication by mold can be expensive. Furthermore, for many cases a range of features, like holes, slots, and pockets, are difficult to incorporate into the part made by the molding process. Therefore, to extend the applications of BMGs in various industrial sectors, it is important to establish sustainable and cost-effective machining processes for successful machining of bulk metallic glasses.

**Figure 1:**
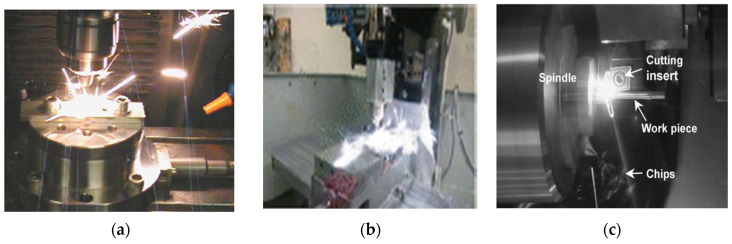
The light emission phenomenon during machining of BMG: (**a**) drilling [3], (**b**) milling [4], and (**c**) turning process [5]. (With kind permissions from Elsevier (**a**,**c**) and Springer (**b**)).

Since the introduction of BMG in 1960 by Klement and Duwez at Caltech [2], a branch of research on BMGs has focused on successfully machining and processing BMGs for various applications. Lack of plasticity and crystallization during machining have created challenges in harnessing bulk metallic glasses, since the crystalline phase can make metallic glasses brittle. In addition, especially for conventional machining processes, metallic glass introduces challenges in reducing oxidization, tool life, and chip formation. One of the signature phenomena in the machining of BMGs is the light emission phenomenon and/or creation of lightning chips, which introduces a fire hazard during machining, as can be seen in Figure 1 [3,4,5]. The machining of metallic glasses is desirable in the manufacturing of electronic components, precision surgical instruments, and micro-electro-mechanical devices. Challenges in the machining of BMGs remain as follows: catastrophic failure, dimensional limitations, and high cost due to expensive raw materials. Crystallization is the main drawback of this metal during conventional mechanical machining because of exposure to stress and temperature variations. Metallic glasses also lack grain size, which is very useful in forming precise shapes. There have been efforts made to improve the characteristics of metallic glass by reinforcing metallic glass matrix composites. Metallic glasses lack the grain boundaries that result in shear bands and are important in determining the plasticity of the material.

Since the first metallic glass research in 1960 [6], there has been extensive research on the development and application of this important material. In recent years, several review papers have been published focusing on various aspects of bulk metallic glass. The origins of bulk metallic glass research have also been discussed, and the evolution of research on the development of various bulk metallic glasses have been reported. Two important aspects or branches of research on bulk metallic glasses have been highlighted, which are plastic-like processibility of BMGs and enhanced fracture mechanics of BMGs. The many potential applications of BMGs in mold and die making have also been highlighted, namely electronics, and the ways in which their properties and processing capabilities made them a suitable option for the proposed applications have been discussed. Kruzic (2016) [7] carried out a review of important mechanical properties of BMGs and identified strategies to improve the mechanical properties of BMGs, with the goal of enhancing engineering applications of BMGs as a structural material. Greer and Ma (2007) [8] reported major challenges relating to material issues in BMG development, and how the structure–properties relationships of BMGs influence their applications. The authors also provided guidelines to resolve major material issues and suggested future research directions on the further development and growth of potential applications of BMGs. In addition to structure–processing–properties research, there has been a significant amount of research on the manufacturability and various fabrication techniques of BMGs and relevant applications. Khan et al. (2017) [9] carried out a review of various production methods for BMGs and the applications associated with those production methods. They discussed various fabrication methods, including liquid and solid-state joining, selective laser melting, and spark plasma sintering, as well micro and nano imprinting methods of BMGs for micro and nano applications. The authors also discussed how the fabrication methods influence corrosion and the magnetic properties of BMGs and presented state-of-the-art research on various magnetic properties of BMGs. The applications of BMGs in various areas including in biomedical devices, electronics, catalysis, fuel cells, and multi-scale mechanical devices have been reported. Although the review focused on various fabrication methods for BMGs, the authors did not consider the machinability of BMGs in their review paper.

There have been a few attempts to gather collective information on the machinability of BMGs. Bakkal (2017) [10] attempted to investigate the machinability of BMGs using various conventional machining techniques, including milling, turning, drilling, and grinding processes. The author mostly summarized his research articles and PhD thesis on various aspects of the machinability of BMGs, including cutting forces, tool wear, and chip formation mechanisms. Zhang and Huang (2019) [11] produced a review of the literature on the micro scale machining of BMGs mostly using three micromachining processes, such as micro-EDM, laser micro-machining, and diamond turning. They discussed various aspects of the machining of BMGs using these processes, including the effects of various machining conditions on the machinability of BMGs and challenges faced during machining. However, the authors did not consider some of the conventional and non-conventional machining processes in their review. In addition, advanced machining processes combining two or more processes in a single setup (i.e., hybrid machining) were not discussed in their review paper. Williams and Lavery (2017) [12] presented a review on laser processing of BMGs, which is considered as one of the most widely used processes for processing bulk metallic glass. The authors considered different aspects of laser processing and fabrication, including laser welding, laser cladding, laser sintering or layer-by-layer additive manufacturing, and surface or sub-surface modification of BMGs using laser. The goal of the paper was to enhance the understandability of property changes in BMGs when processing using various laser processing or fabrication techniques.

Although several studies have taken the initiative to compile research on various aspects of structure, processing, properties, and applications of BMGs, there have been few efforts to review the machinability and various fabrication techniques of BMGs. It is important for the readers to understand the current state of research on the machinability of BMGs as well the challenges faced when employing different machining techniques while machining this difficult-to-machine material. Although a few review papers have been published in recent years on the micromachining and laser processing of BMGs, a comprehensive review covering all aspects of multi-scale conventional, non-conventional, and hybrid machining of BMGs is missing. In this review paper, research works in the field of the machining of bulk metallic glasses, covering various aspects of machinability in both conventional and non-conventional machining, have been included. Both macro and micro scale machining of BMGs are covered in this review paper. Current challenges and unsolved research questions on areas of both conventional and non-conventional machining of BMGs have been identified and discussed. In addition, some potential solutions for resolving challenges in the machining of BMGs and future research directions have been presented. The novelty of this study lies in identifying challenges in machining BMGs and correlating the challenges with their structure, processing, and properties, and then discussing potential solutions in terms of selecting advanced non-traditional and hybrid machining processes. The paper also presents critical analysis of existing literature and offers suggestions for future and unexplored research areas on the machining of BMGs.

## 2. Brief Overview of Structure, Processing, and Properties of BMGs

**Figure 2:**
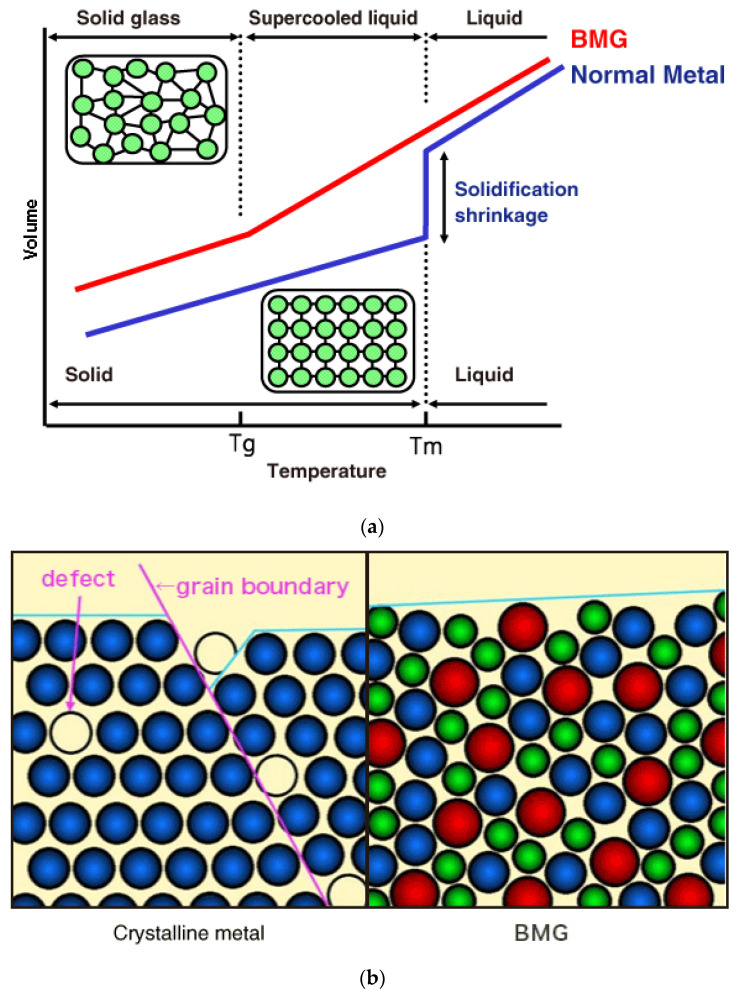
Schematic diagram showing the traditional crystalline microstructure vs. the amorphous microstructure of the BMGs; (**a**) solidification curves of normal metal vs BMG, and (**b**) crystalline microstructure of normal metal vs amorphous microstructure of BMG; Microstructure of normal metal includes grains of a single color indicating one element, whereas microstructure of BMG includes multi-color grains indicating composition of BMG with multiple elements [13]. (Copyright: Orbray.com; open access).

Traditional metals or their alloys have an ordered, long-range crystal structure due to a slow cooling rate, which provides sufficient time for atoms to rearrange by themselves in an orderly manner. On the other hand, metallic glasses lack this orderly atomic arrangement due to fast cooling (10^6^ K/s), which results in insufficient time for atoms to rearrange into a crystal [13]. However, in real life, achieving this slow cooling rate of 10^6^ K/s is not very easy; therefore, bulk metallic glasses, which are usually fewer millimeters thick and can be manufactured using cooling rates below 10^6^ K/s, have become popular. Generally, each alloy has its own critical cooling rate for generating a non-crystalline structure, which also depends on the fabrication techniques. Glass forming ability (GFA) is the term used to indicate the ability to form BMG at a certain cooling rate. At a cooling rate above the critical value, components can combine together to produce a non-crystalline alloy by freezing any random structure. Figure 2 shows the schematic representation of the microstructural differences between BMGs (amorphous microstructure) and traditional metals and alloys (crystalline microstructure) [13].

**Figure 3:**
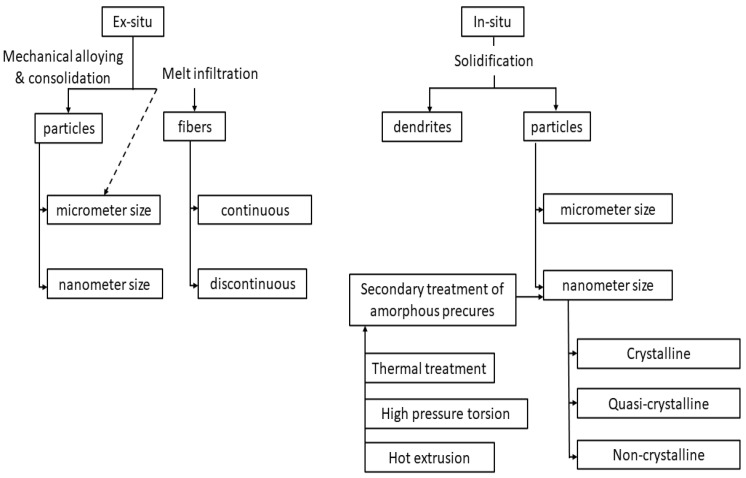
Various manufacturing and processing techniques for developing bulk metallic glasses [28]. (With kind permission from Springer).

Solid-state amorphization is one of the most common ways of manufacturing BMGs, either using mechanical alloying or powder metallurgy [14,15]. Rapid cooling of the liquid-state material is exploited, using various methods such as mold casting, atomization, or melt spinning [16,17,18,19]. In the case of powder metallurgy, powder particles obtained from atomization techniques must be combined together using hot press, extrusion, or other similar technologies [15,20,21]. On the other hand, the solidification technique facilitates a simple and direct route of manufacturing BMGs. In addition, it is possible to manufacture BMG composites using either solidification or mechanical alloying. Partial devitrification of amorphous precursors either by thermal treatment or by plastic deformation can also be used for composites [22,23,24,25,26,27]. Figure 3 provides a glimpse of different processing techniques of BMGs [28].

**Figure 4:**
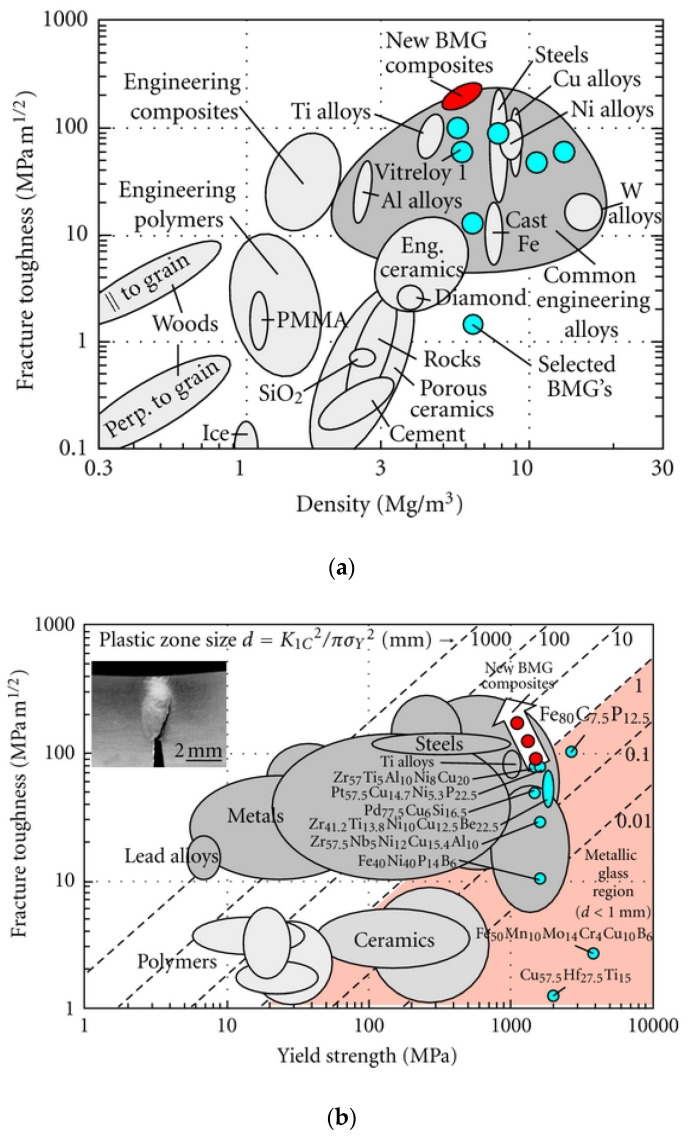
Fracture toughness of various materials against their (**a**) density and (**b**) yield strength, comparing BMG and its composites with other materials [6]. (Copyright: Hindawi, Open Access).

Due to its unique structure, unlike crystalline materials, BMG exhibits very different mechanical properties. Although both BMG and crystalline materials possess a similar magnitude of elastic modules in the same composition, BMG exhibits a higher magnitude of strength at room temperature and experiences no strain hardening. On the other hand, the plastic deformation of BMG that occurs through homogenous plastic strain depends not only on normal stress but also on shear stress. In addition, BMG exhibits Newtonian flow when exposed to low stress and increased temperatures [29]. Elastic modulus and glass transition temperature influence the hardness and fracture strength of BMG. Therefore, BMG with maximized properties of strength, toughness, and plastic deformation can be achieved by optimizing elastic properties like Poisson’s ratio or shear to bulk modulus ratio [30]. Figure 4 compares the fracture toughness of BMG and its composites with respect to other materials such as polymers, ceramics, metals, and alloys [6].

**Figure 5:**
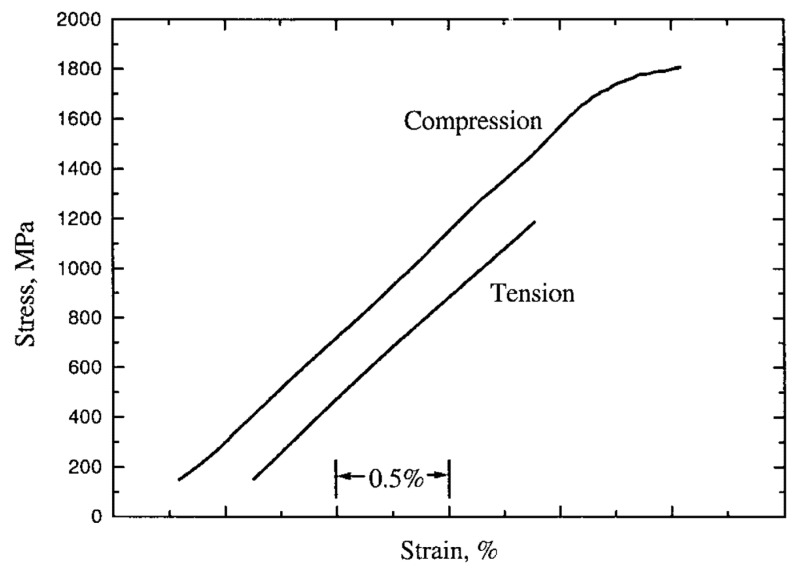
Typical tensile and compressive stress–strain curves of Zr_57_Nb_5_Cu_15.4_Ni_12.6_Al_10_ bulk metallic glass [33]. (With kind permission from Springer).

BMG shows no plastic deformation under tensile loading, however, a small amount of ductile behavior under compressive load is common due to the extra constraint resulting from the large localized plastic strain of the shear band. Therefore, large localized plastic strain, slipping, and intersection of multiple shear bands facilitate the plasticity of BMG under compression [31]. On the other hand, tensile loading causes the failure of BMG by shear rupture through single shear band [32]. Figure 5 shows the stress–strain behavior of BMG under tension and compression [33].

**Figure 6:**
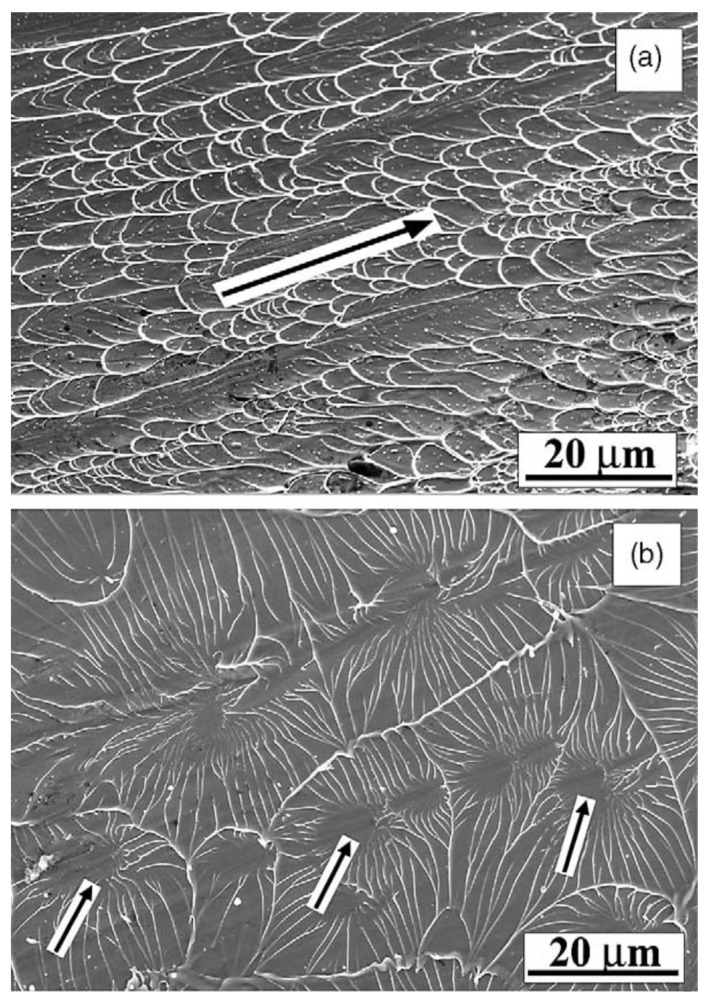
Representative fracture surfaces of Zr_59_Cu_20_Al_10_Ni_8_Ti_3_ under (**a**) compression and (**b**) tension; the fracture direction is shown by arrows [34]. (With kind permission from Elsevier).

In addition, fractured surfaces show some dissimilar behavior under tensile and compressive loading conditions. Fractured surfaces, due to compression load, exhibit periodic bands in the fracture direction, along with uniform vein-like structures inside the bands due to the dominance of shear stress [34,35]. On the other hand, fractured surfaces under tensile stress exhibit a combination of veins and radial cores due to the dominance of normal stress. There is also a possibility of having cored structure formation during compression stress, which might get obscured [36]. Figure 6 shows the signature signs of typical fractured surfaces obtained in compression and tensile loading [34].

The brittleness characteristics of BMG are also correlated with the modulus of elasticity and Poisson’s ratio [36,37,38]. Based on micro hardness experiments, Chen et al. [39] recommended the critical value of Poisson’s ratio as being about 0.32. Any value above this results in plastic deformation. In addition, any value below 0.41 for universal critical G/K (shear modulus/bulk modulus) gives rise to plastic deformation. In fracture mode I testing on Fe-based amorphous solids, using notched toughness samples, fracture energy was found to be correlated with changes in chemistry induced by Poisson’s ratio and ratios of shear modulus/bulk modulus. Fracture mode II failure under compression also relates strain energy to the similar parameters [40,41].

Under cyclic loading, BMG shows fatigue degradation due to initiation of shear localization. As per the literature, the fatigue endurance limit can be as low as 9% [42] of its tensile strength to as high as 30–35% of tensile strength [43,44,45,46]. Fatigue fracture of BMG exhibits four regions, comprising the initiation of crack zone, stable crack growth zone, unstable crack growth zone, and a melting zone. Stress concentration or preexisting defects are more likely to be responsible for initiating the damage in the case of fatigue fracture. Zr-based BMGs show better fatigue performance compared to Cu-based BMGs in some cases, due to the presence of nanocrystalline particles [47,48,49,50]. BMGs are also vulnerable to surface defects like notch, which can reduce strength properties and ductility [51]. Dynamic loading tests reveal lower hardness values as well as severe deformation when compared to static loading conditions. While widely spaced semicircular shear bands are observed for higher indentation rate, lower indentation rate causes closely spaced semicircular shear bands. Stress state variation, strain rate, and temperature are more likely be responsible for this difference in shear band patterns. Additionally, higher strain rate promotes free volume migration as well as nano-void formation, due to the condition of adiabatic heating, and contributes to eventual softening [52]. Regarding the toughness of Zn-based BMGs, dynamic fracture toughness exhibits a four to six times greater increment in value compared to quasi-static regime. Inertia and thermal softening affecting the crack nucleation process are considered responsible for this rapid increment change [53]. However, an insignificant effect on toughness is also observed for a change in loading rate by Lewandowski [54]. This study shows that the mechanical properties of BMG are largely controlled by the internal structure of BMG. Various treatments, such as annealing or controlling cooling rates, may contribute in varying the chemical and topological atomic order of BMG, resulting in various mechanical properties [55,56].

BMG exhibits lower density compared to crystalline materials in an order of 0.5–1%. However, BMG shows a density value close to crystalline materials when it is in a crystalline state, meaning it also experiences closely packed atomic structure [57,58]. The thermal expansion coefficient of BMG appears to be close to the value it would have if it were to be in crystalline state. The non-crystalline structure of BMG facilitates lower electrical conductivity (high resistivity) compared to the crystalline one, and it exhibits moderately negative temperature dependency despite its metallic bonding [59,60,61]. The specific heat of BMG also exhibits a similar value if it is in a crystalline state and increases along with the increase in temperature [62,63]. Regarding magnetic properties, iron- and cobalt-based BMGs exhibit low coercive force and thermal losses, higher saturation magnetization, and high permeability, and they are known as magnetically soft materials [64]. In addition, BMGs show an excellent resistance towards corrosion and passivation due to their single-phase structure, which lacks the presence of lattice defects such as dislocation and grain boundaries. This special higher resistance property makes it possible for BMGs to be applied as biomaterials [65].

Microstructure and mechanical properties influence the machinability of a material. The amorphous microstructure, high hardness, and strength of BMGs make the machining of BMGs challenging. As a result, many researchers have focused on investigating the machinability of difficult-to-cut BMGs using various machining processes. In the following sections, brief overviews of various research studies on the machining of BMGs will be discussed. The machining processes can be broadly classified as conventional and non-conventional machining processes and, hence, research studies on the machining of BMGs using various machining techniques reported in the literature have been discussed in the following sections under two major headings of conventional and non-conventional machining of BMGs.

## 3. Conventional Machining of Bulk Metallic Glass

### 3.1. Milling of Bulk Metallic Glass

Milling is a well known machining process capable of producing complex 3D parts. Conventional milling processes and micro milling processes are quite similar, and the differences are mainly attributed to differences in the size of interest. Micro milling can produce 3D components at a micro scale while also providing good surface finish and high accuracy. In addition to its attractive features, micro milling introduces challenges when it comes to tool life. Working with micro-operations requires thorough study of micro milling process parameters, such as spindle speed, feed rate, and tool material and radius. Several studies have been conducted on various types of bulk metallic glasses using micro milling.

Wang et al. (2020) [66] investigated the machinability of a Zr-based BMG (Zr_41.2_Ti_13.8_Cu_12.5_Ni_10_Be_22.5_) using the milling process with a coated tungsten carbide cutting tool. The machinability was analyzed, using the tool wear and remaining tool life, while machine a certain length of BMG compared to an aluminum alloy. A comparison of surface roughness and cutting forces between BMG and the aluminum alloy was made. It was reported that the cutting forces were higher in the machining of BMGs compared to the aluminum alloy, mainly because of the high hardness and strength of the BMG. It was found that there were some crystallizations at the BMG machined surface, however, with careful parameter selection in dry machining, the amorphous phase could still be maintained. The tool wear mechanisms in the machining of BMG were found to be different than those for machining aluminum alloy. The cutting tool was found to have a remaining tool life after milling about 300 mm length, and the most common tool wear mechanism observed during the machining of BMG was chipping due to the high hardness of the BMG. The surface roughness could be reduced down to 0.06 um during micro milling of BMG using finishing regime parameter selection.

Xie et al. (2017) [4] investigated the behavior of zirconium-based BMG under micro machining using varying spindle speeds. It was found that at the beginning of the micro milling process no light emission was seen. However, light started to appear as the machining continued at a higher cutting speed due to heat accumulation. A high feed rate and spindle speed produced light with extensive intensity. It was also found that constant spindle speed with high feed rate changed the structure of BMG from amorphous to crystalline. Surface cracks appeared because of the workpiece melting and solidifying. Surface morphology was disrupted as the heat was inducted and collected in the material. It was reported that micro milling of BMG with a spindle speed below 30,000 rpm and feed rate increments up to 10 μm/flute produced no crystallization phase.

One of the alternatives to the physical study of the machining of BMG is to conduct finite element analysis (FEA). FEA, through the use of a software package (Deform 3D Version 6.1), reduces expensive experiment procedures. Karaguzel and Bakkal [67] conducted FEA with the commercial software DEFORM 3D (Version 6.1) by modeling cutting tools to investigate the machinability of Zr-based BMG. During the experiments, a slot milling process was performed. Cutting forces were collected for four different feed rates, while cutting speed and depth of cut were held constant. It was reported that a lower feed rate resulted in a reduction of chip thickness. It was discovered that there was a lack of various frictions in the simulation, and the roundness of tools with smaller chip thickness was not taken into account; therefore, low feed rate simulation could not provide comprehensive results. It was reported that use of simulation software could only provide a general understanding of the behavior of BMG under simple conditions. However, to be able to recreate micro machining conditions and the actual behavior of the machined BMG, a real-life investigation through carefully designed experiments must be carried out.

Ion milling is widely used to produce micro/nano scale specimens for transmission electron microscopy (TEM). TEM is used to study the microstructures, lattice structure, and phase transformation of a material by passing electrons through the specimen. Gu et al. (2010) [68] machined several TEM specimens of Zr-based metallic glass using ion milling under different gun currents, gun voltages, and incident angles. It was found that the heat produced during ion milling for TEM specimens was higher than the glass transition temperature of Zr metallic glass. The specimen produced using ion milling had substantial crystallization and the highest nominal temperature induction. The use of nitrogen for ion milling showed non-uniform local heating. Overall, ion milling could result in very high temperatures during the machining of BMGs and caution must be exercised when preparing TEM specimens using ion milling.

**Figure 7:**
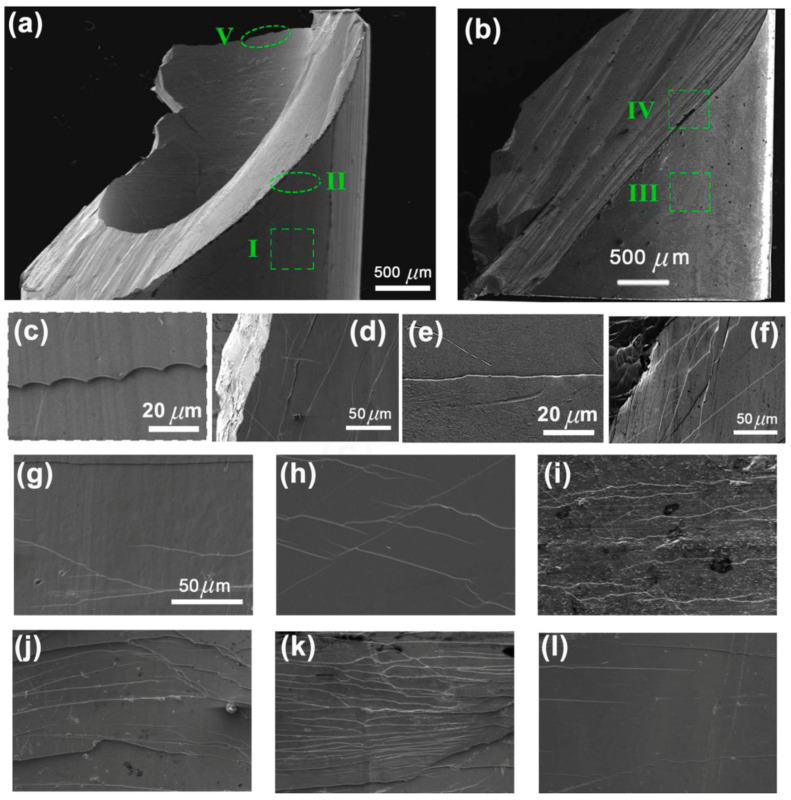
Different views of intact and machined BMG specimens; (**a**) intact, (**b**) machined, (**c**–**f**) close up views of different positions, (**g**–**l**) inner wall of machined BMG specimens [69]. (With kind permission from Elsevier).

Gong et al. (2016) [69] aimed at improving the mechanical performance of Zr-based BMG by milling through holes on the BMG specimens to generate shear bands. Specimens with machined artificial holes, especially with diameter ratios of hole to rod of 3:5 and 7:10, demonstrated better plasticity (2% and 4.5% more, respectively) when compared to the original cast specimen, which exhibited brittle fracture behavior with almost no plasticity. It was theorized that shear band generation stimulated by the inner through hole creates a blocking effect on the propagating shear band much like a dislocation mechanism, as shown in Figure 7, and therefore enhances the mechanical properties [69].

Maroju et al. [70] performed high speed milling of Zr-based BMG to investigate surface microstructure and progressive tool wear. The effect of coolant on tool wear was also explored. The milling of BMG introduced a crystallized phase as well as oxidation of the surface. Due to continuous light emission, the material continuously melted and redeposited, which further resulted in voids on the machined surface. It was resolved that adhesion wear was the dominant factor in flank tool wear.

Ray et al. [71] fabricated micro-channels over Zr-based BMG using a micro-milling process and looked into the effects of process parameters on the quality of micro-channels. It was found that the average micro-channel width decreased with the increase in the axial depth of cut, while the increase in depth of cut resulted in higher dimensional inaccuracy. Furthermore, the channel width was found closer to the desired values when a higher feed rate was applied. The surface roughness was independent of the axial depth of cut and increased with the increase in spindle speed.

In another study by Maroju and Jin (2018), dimples were fabricated over Zr-based BMG by an inclined high-speed milling process with a single flute PCD tool [72]. The effects of vibration assistance on process performance and material deformation were also explored. Inclined milling produced high stress, which further resulted in tool tip fracture. It was found that chip formation along with tool wear were responsible in determining the quality of dimples. The hardness played a vital role in tool wear, while the fracture toughness determined the chip produced during machining. On the other hand, uniform dimples were fabricated over the machined surface when vibration-assisted inclined milling was adapted compared to conventional inclined milling process. This was mainly due to the preserved tool geometry and reduced chip load. In addition to that, the vibration-assisted inclined milling generated a hybrid micro-texture within a single dimple and the vibration noticeably affected the chip formation.

### 3.2. Turning of Bulk Metallic Glass

Turning is a popular and a vital machining process for machining cylindrical shafts and components. Both macro and micro turning work similarly by rotating the work piece and introducing tool bits to cause a desired cut. The mechanical and thermal properties of a material play an essential role in determining the machinability during turning. Cutting forces, surface and sub-surface integrity, chip formation, and tool life are important features to consider while machining by turning, and they require a thorough study of process parameters to produce the desired surface finish or sub-surface microstructures.

**Figure 8:**
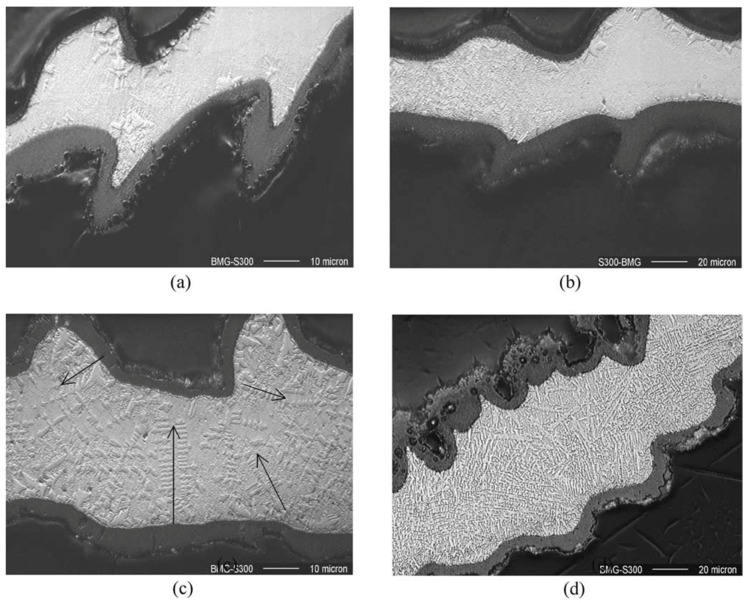
Images of BMG chip morphology showing (**a**,**b**) crystallization and oxide layers, (**c**) dendritic branch, and (**d**) fully crystalline layer [5]. (With kind permission from Elsevier).

**Figure 9:**
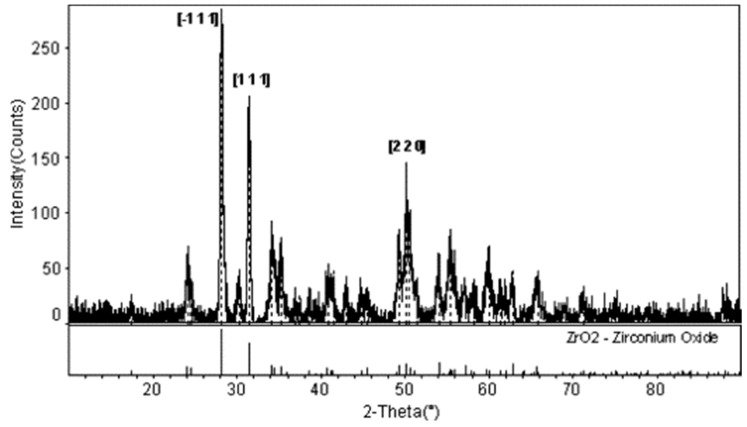
X-ray diffraction analysis of the chips showing crystallization and indicating presence of ZrO_2_ at 0.76 and 1.52 m/s. [5]. (With kind permission from Elsevier).

Bakkal et al. (2004a) [5] used a turning process to machine Zr-based BMG to study the oxidation and crystallization effects on the chips and the machined surface. The research showed that the light emission during machining and the heat generated at a high cutting speed led to oxidation of the chips. It was found that a low cutting speed produced no crystallization on the machined surface or the chips. Microstructural analysis of the chips clearly indicated a thin layer of crystallization at the periphery of the chips at a higher cutting speed. Figure 8 shows the transition from amorphous to crystalline structures of chips when machining at a higher cutting speed (1.52 m/s). After polishing the surface area of chip, a crystallization pattern with dendritic formation was observed. Due to oxidation thickness, it was difficult to find the diffraction pattern at a higher depth. The thickness of the oxide layer was about 5–10 μm. The test revealed that high temperature machining leads to oxidation of Zirconium, which resulted in crystallization. X-ray diffraction (XRD) analysis, as shown in Figure 9, revealed a diffraction pattern similar to ZrO_2_, thus confirming oxidation of the chips.

In another study, Bakkal et al. (2004b) [73] examined the machining behavior of ZrTiAlCuNi metallic glass under 0.76 m/s and 1.52 m/s cutting speeds with TiN-coated WC-Co insert. Light emission was frequently observed during the machining of BMG at a higher cutting speed, especially during the fracture while machining because of the exothermic oxidation reaction. It was found that as the cutting speed increased, the elastic modulus and hardness of the material increased substantially. Similar to past experiments, the diffraction peaks of this BMG matched ZrO_2_. In the chip morphology, shear lamella was observed, which was separated by shear localization. The variation in chip morphology at different cutting speeds reveals increasing crystallization. It was reported that during the chip removal process, viscous flow and crystallization occurred.

**Figure 10:**
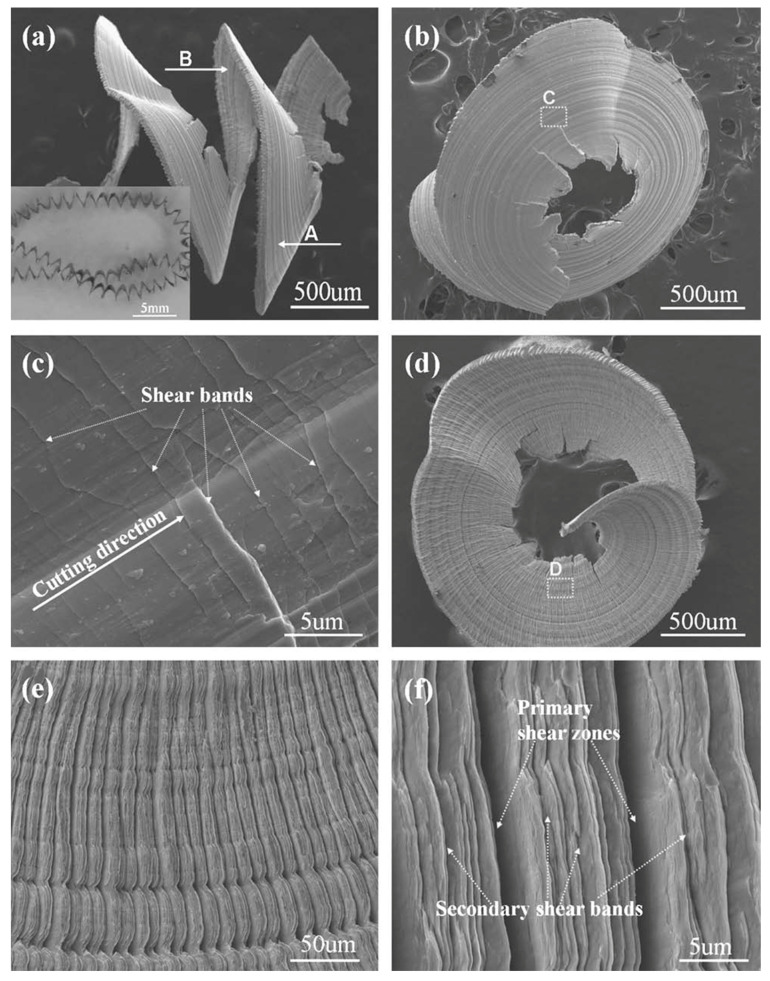
Chip morphology of BMG showing shear bands and primary shear zones indicating crystallization after machining; (**a**) a typical twisted continuous chip, (**b**) chip surface marked by A in (**a**,**c**) details of area C in (**b**,**d**) free surface of chip marked by B in (**a**,**e**,**f**) lamellar structure marked by D in (**d**) at two magnifications [74]. (With kind permission from Elsevier).

Jiang and Dai (2009) [74] investigated the turning of ZrTiCuNiBe BMG to study the lamellar chip formation mechanism and compare theoretical assumptions with experimental results. It was observed that production of lamellar chips was due to thermal instability resulting in shear localization, which is explained by the bifurcation phenomenon. Chip images can be found in Figure 10, which displays the formation of shear bands. High free volume concentration with a high Poisson ratio resulted in a reduction in continuous lamellar chip formation. High cutting speed, low heat flow coefficient, and high depth also produced lamellar chips. It was found that heat flow coefficient is a major factor for determining the formation of lamellar chips. By reducing heat flow, lamellar structure can be obtained. It is suggested that thermal instability and free volume instability be controlled while machining metallic glass.

**Figure 11:**
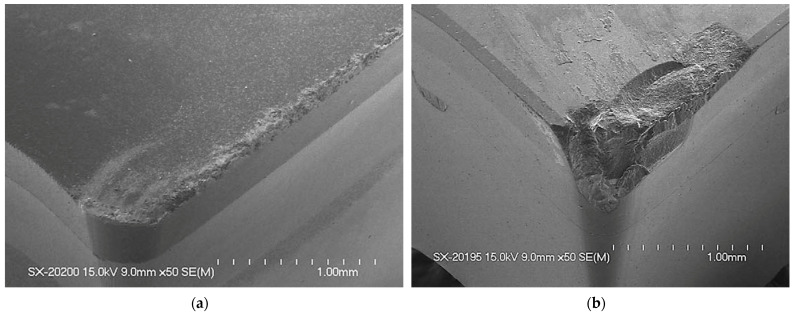
SEM images showing tool wear at the tool nose and cutting edge of the (**a**) PCBN tool and (**b**) PCD tool after machining BMG [75]. (With kind permission from Elsevier).

Bakkal et al. (2004c) [75] compared the machinability of Zr-based BMG with aluminum 6061-T6 and AISI 304 stainless steel by analyzing chip morphology, surface roughness, and tool wear. High cutting speed and low thermal conductivity were found to trigger light emission and oxide formation on chips. Serrated chip formation with an adiabatic shear band was seen if there was no light emission. It was found that Zr-based BMG had better surface finish than the other two alloys. Tungsten carbide tools with PVD coating (WC-PVD) generated no oxidation and light emission, which could have been due to the high rake angle of the tool. The PCD tool did not generate light emission either because of its high thermal conductivity. Other tools used during machining generated light emissions. The cutting force was seen to be reduced due to thermal softening at high speed. The tool wear was found to be a major problem during machining BMG; however, BMG had better surface finish, which implies that BMG has optical and photonic applications. Figure 11 shows the tool wear suffered by the PCBN and PCD tools after machining BMGs [75].

**Figure 12:**
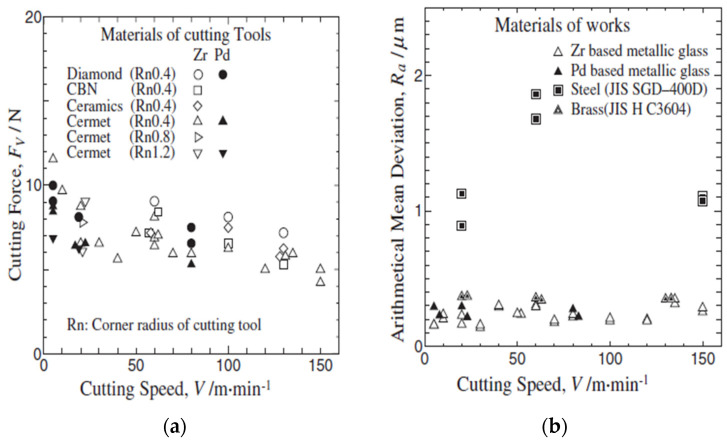
(**a**) Cutting forces vs. cutting speed using different tools; (**b**) arithmetic mean deviation on the surfaces of the BMG, steel, and brass workpieces using the cermet tool tip [76]. (Copyright: J-STAGE, Open Access).

Fujita et al. (2005) [76] compared the machinability of Zr- and Pd-based BMGs to obtain the effects on surface finish and cutting force from several tools. The tool tips used in this study were diamond, CBN, ceramics, and cermet. Results were compared with crystalline alloys, steel, and free-cutting brass. Feed rate and depth of cut were constant throughout the experiment. It was found that with cermet tool, cutting force generated during the machining of BMG was half of that of steel at slow cutting speed, whereas with diamond tool the cutting force was almost identical at higher cutting speeds. The cutting force was observed to be higher for Pd-based BMG when machining with the cermet tools. With a diamond tool tip, the cutting force was the lowest on Zr BMG, as shown in Figure 12a [76]. Both diamond and cermet tips were found to be effective in the reduction in cutting force on Zr BMG. Despite having variable cutting speed, surface roughness for both BMGs was found to be similar. Figure 12b shows how the machined surface roughness varied for different workpiece materials for machining with cermet tools [76]. It was found that surface roughness was lower for both BMGs in comparison to steel and free-cutting brass, indicating its capability of generating a smoother and shinier surface after machining. It is also reported that nose tip radii and surface roughness are inversely related. Lack of built-up edges during lathing was found to lead to better surface finish with BMGs. Chip formation was highly affected by maximum shear stress because of BMG’s amorphous structure.

Wang et al. [77] investigated the influence of different machining parameters on the surface finish of Zr-based BMG. It was found that, when using a single crystal diamond tool, low surface roughness was achieved with high feed rates and spindle speed. A fold-like pattern was observed on the surface of the generated chips, which could have been caused by movements from the diamond tool. It was also found that a higher feed rate generated better surface finish, but not without compromising the stability of the tool. A larger depth of cut also resulted in sinusoidal grooves, while high feed rate resulted in irregular grooves on the surface. Irregular grooves could have been caused by layer softening at a high feed rate during the machining of BMG.

Ding et al. (2020) [78] investigated the chip formation mechanism during orthogonal cutting of a Zr-based BMG and discussed the machining behavior of BMG by analyzing the chip formation mechanism, cutting speed, and surface and sub-surface integrity. The effect of different cutting conditions on the stress, strain, associated temperature generation at the primary shear zone, and energy consumption during chip formation have been discussed. It was reported that the orthogonal cutting of BMG generated serrated chips and the degree of serration, strain, and stress generation increased with the increase in cutting speed, due to an increase in friction at the tool–chip interface. It was suggested that the measurements of signature parameters of serration from the chip morphology could be used to analyze the material and machining behavior of BMG. In addition, these can provide important information regarding energy consumption during machining. An interesting phenomenon of dimple generation at the root of the chips was reported, which along with the ductile tearing causes separation or removal of chips from the BMG.

Xiong et al. [79] conducted a series of turning experiments, varying cutting parameters to look into the machinability of Pd_40_Ni_10_Cu_30_P_20_. The assessment considered the quality of the machined surface, chip morphology, and tool wear. Employing a natural diamond tool for cutting, it was observed that the Pd-based bulk metallic glass (BMG) exhibited excellent machinability, resulting in a machined surface roughness of approximately 3 nm. Along the tool path on the outer circular region of the machined surface, irregular micro/nanostructures were identified, significantly impacting surface roughness. In addition to that, the cutting heat resulted in softening of the workpiece material, flattening the tool marks under surface tension and leading to an improvement in surface quality.

**Figure 13:**
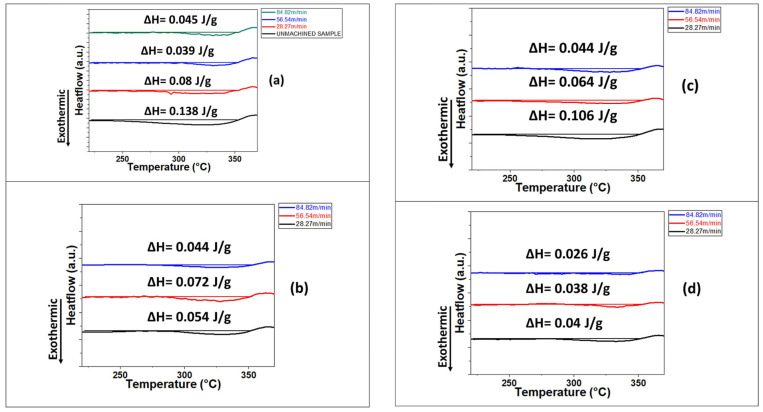
DSC graphs of machined BMGs showing enthalpy of relaxation and estimation of free volume for different experimental conditions; (**a**–**d**) indicates varied feed rate of 10 μm, 20 μm, 30 μm, and 40 μm per rev. [80] With kind permission from Elsevier.

In a recent study by Dhale et al. (2022), the micro-turning of Zr-based bulk metallic glass was executed to examine variations in the machined sub-surface [80]. As mentioned earlier, plastic deformation of metallic glass can be tuned by the formation of shear bands. Material experiencing external shear stress may compel the collective rearrangement of atoms to induce a fine zone of shear transformation (STZ), along with a change in the free volume of the concerned material. It is reported that free volume is a precondition for STZ to take place and larger free volume is correlated with the triggering of a large amount of STZ. Shear bands form with the accumulation of STZ [81,82,83]. A study by Dhale et al. (2022) focused on the reduction of free volume and STZ volume in the machined sub-surface as a result of machining. It was noted that the micro-turning process resulted in the hardening of the sub-surface, with this effect being confined to a depth of up to 20 microns from the machined surface due to annihilation of free volume. In such a case, a nano indentation technique can be implemented to measure this hardened surface, along with DSC curves, which are capable of experimentally measuring the decrease in free volume due to the structural relaxation of an amorphous solid. Based on the direct correlation between enthalpy at a glass transition temperature and free volume, relaxation enthalpy measured using DSC curves can provide a good indication of free volume. As shown in Figure 13, the DSC curve area under the machined hardened surface appears to be less than the DSC curve area under the unmachined surface, representing the presence of reduced free volume for a machined surface compared to unmachined one. The changes in enthalpy were quantified, revealing a lower value in the hardened region and therefore signifying a reduced free volume content in the machined surface. Thus, the change in free volume associated with a hardened machined surface can be measured and contrasted with that of an undeformed/unmachined surface.

### 3.3. Drilling of Bulk Metallic Glass

Drilling is a machining process that utilizes drilling tools to machine holes in a work piece. Drilling is extensively used to produce parts with complex shapes and high dimensional accuracy. The end result of drilling is highly influenced by parameters such as spindle speed, feed rate, tool material, and coolants. Compared to other traditional machining processes, fewer studies have been performed on the drilling of bulk metallic glasses, which opens up possibilities for more applications of the drilling process in metallic glasses.

**Figure 14:**
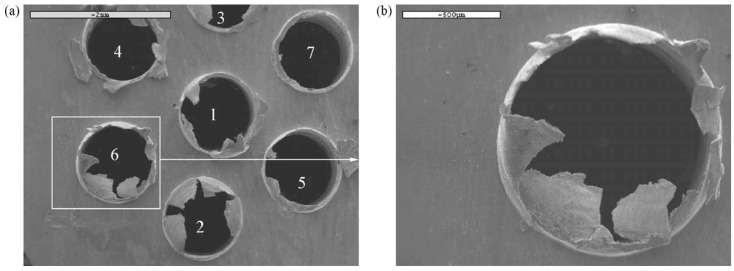
SEM images showing crown-shape exit burr after drilling holes in BMG using a HSS tool at 10 mm/min feed rate; (**a**) general view, and (**b**) magnified view of burrs at the edge of drilled holes; 1–7 just show numbers of holes machined; Scale bars in the figure are 2 mm for (**a**) and 500 μm for (**b**) [3]. (With kind permission from Elsevier).

Bakkal et al. (2005) [3] investigated the light emission phenomenon, surface morphology, and burr formation upon drilling of BMG. The alloy used in the study was Zn-based BMG. As a source of comparison, AISI 304 stainless steel was also used, and the drilling was performed under the same conditions. The cutting tool materials used were M7 high speed steel and tungsten carbide in cobalt matrix (WC-Co). Drilling experiments were conducted under varying feed rate, spindle speed, drill size, cutting fluid, and tool material to investigate the effects of these variables on the drilling performance of BMG. It was found that reducing feed rate and increasing cutting speed increased the formation of oxidation when the drilling operation was carried out without any use of coolant. This is very similar to the phenomenon that was observed in the milling and turning of BMGs, as discussed in previous sections. During experimentation, the highest intensity of light was seen when a drill diameter of 2 mm was used. No light emission was observed with the tool with higher thermal conductivity as the heat energy is reduced in the chip. The contact between BMG and tool increases as they rub against each other at high speed, thus leading to exothermic oxidation. Different types of chips were observed with changes in parameters. Some of the chips were short ribbons, long ribbons, fan shaped, powdered, long spirals, and long tangled ribbons. It was found that use of cutting fluid and high feed rate promoted long ribbon chips. Long tangled ribbon chips were formed with higher spindle speed, while long spiral chips were formed with slow feed rates and the use of a WC-Co drill. Burr formation is a critical component of drilling, as it was found to be difficult to machine holes in BMG without burr formation. High feed rate seemed to reduce the size of burr in both entry and exit areas. Figure 14 shows the burr formation around the edge of micro-holes machined in BMG using the drilling process [3].

**Figure 15:**
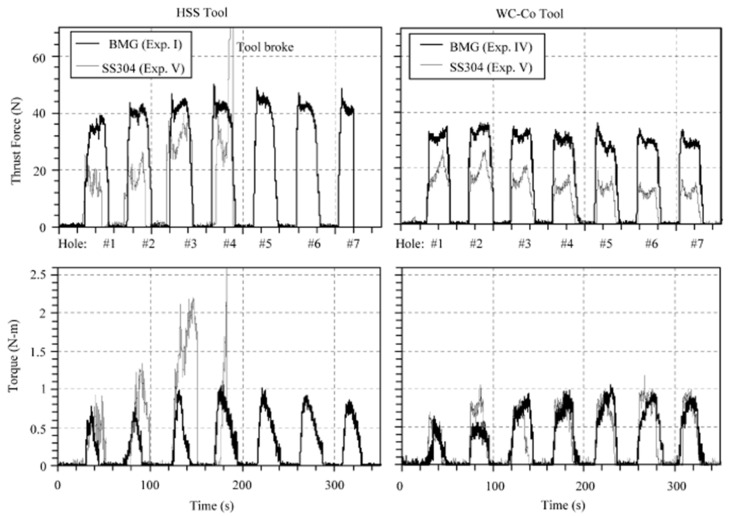
Thrust force and torque signals during machining of BMGs using HSS and WC-Co tools at a feed rate of 5 mm/min and tool rotational speed of 10,000 rpm [81]. (With kind permission from Elsevier).

**Figure 16:**
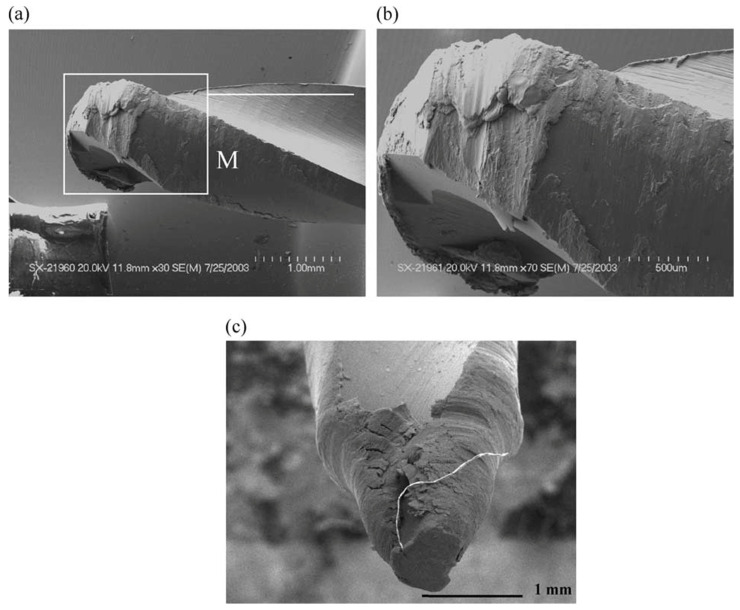
SEM images showing extreme tool wear and plastic deformation of cutting tools during machining of BMG using a 2 mm diameter HSS tool and a feed rate of (**a**,**b**) 10 mm/min and (**c**) 2.5 mm/min [84]. (With kind permission from Elsevier).

Bakkala et al. (2005b) [84] investigated the machinability of Zr-based BMG by drilling at various speeds and compared the machinability of BMG with that of 304 stainless steels. During the procedure, thrust force, torque, and tool wear were investigated. Tool wear was found to be a crucial factor that determined the quality of drilled hole. Tool wear can result in damage of the part and contribute to damage of the machine tool. Two different drill bit materials were used, HSS (high speed steel) and WC in cobalt matrix. It was observed that at lower feed rates, use of HSS produced plastic deformation. Due to high temperatures generated during the drilling of BMG, tool wear was accelerated. Tool temperature also increased with the time of cut. It was found that the spindle speed does not affect the thrust force and torque generated during drilling. Significant tool wear was seen with high speed, while low speed resulted in built up edges in the work material and damaged the drill bits because of the extremity in cutting force. The WC-Co tool resulted in better plowing of the work material with less wear compared to the HSS tool. The HSS tool had more wear and was deformed easily in comparison to the WC-Co tool. Figure 15 shows a comparative analysis of cutting forces generated during machining of BMG and SS304 steels with HSS and WC-Co tools [81]. It can be seen that, irrespective of tool material, the cutting forces generated during the machining of BMG is higher. In addition, the HSS suffered more cutting forces compared to the WC-Co tools, which also explains the higher tool wear associated with the HSS tool during drilling of BMG. Figure 16 shows extreme plastic deformation of the HSS tool, losing its full cutting geometry after machining BMGs [84].

Zr-based metallic glass was machined using longitudinal-torsional ultrasonic-assisted drilling (LTUAD) by Gao et al. [85] The effectiveness and feasibility of producing holes were further compared with that of the holes prepared using a traditional drilling process. It was found that the number of pits, along with the exit round cap, was noticeably reduced when the LTUAD process was employed compared to conventional drilling, improving the holistic quality of the holes manufactured. Furthermore, the mean thrust force was decreased by a maximum of 29.12% in the case of LTUAD when machining holes in BMG.

### 3.4. Grinding of Metallic Glass

Grinding is the most popular abrasive finishing process used for machining hard and brittle materials. In addition to having the capability of machining difficult-to-cut materials, grinding is a common finishing technique used in metal machining industries for other materials as well as BMG. Being a finishing process, the material removal rate of the grinding process is lower compared to other traditional machining processes. As BMGs are known as difficult-to-machine materials, abrasive finishing processes have become a popular method for generating a final surface finish on BMGs.

Bakkal et al. (2015) [86] investigated the machinability of Zr-based bulk metallic glass (Zr_52.5_Ti_5_Cu_17.9_Ni_14.6_Al_10_) using grinding processes. Their study focused on the grindability of materials using a CBN wheel as well as conventional aluminum oxide wheel; the effect of grinding parameters such as feed rate, cutting speed, depth of cut, and type and size of abrasive were considered. Their study indicated that a higher feed rate resulted in larger tangential as well as normal forces. This result can be explained with the fact that higher feed rate results in a higher material removal rate. Higher feed rate also comes with larger chip thickness, which in turn causes reduced ploughing action. Consequently, the specific energy for ploughing, as well as total specific energy, falls. It was reported that the CBN wheel offered stable grinding process for BMGs under the circumstances of a lower feed rate of 1000 mm/min and 2000 mm/min. Depth of cut variation also resulted in similar behavior to the feed rate. In addition, a smaller grit size of the grinding wheel provided smaller normal and tangential forces compared to larger grit size while grinding at the same depth of cut. The grinding force experienced by the conventional aluminum oxide wheel was lower than the CBN wheel at the beginning of the process, however, it exceeded the grinding force of the CBN at the later stage of grinding. The surface analysis of ground BMG shows some initiation of crack regions parallel to the grinding direction, which might be the result of stress-induced volume generation as well as non-uniform deformation. Some ploughing regions as well as micro-fractured areas near the end of grinding groove were also visible. Apart from this, evidence of oxidation due to the presence of Zr and Ti were present, too. Examination of chips adhered to the ground surface confirmed that the material removal took place in a ductile manner. These chips exhibited a lamella structure, along with a different shape and size resulting from adiabatic shear band formation. All this information suggests that ductile chip formation and ploughing mechanisms are the main reasons behind the material removal mechanism during grinding of BMG.

On another study, Serbest et al. (2011) [87] investigated a similar BMG material using a CBN wheel. As per their observation, grinding force showed a decreasing trend, as the specific grinding energy increased along with the increase in cutting speed. This increased specific grinding energy can be explained by the reduced uncut chip thickness, which results in increased sliding and ploughing grinding energy. Their study also demonstrated reduced surface roughness with the increased cutting speed.

**Figure 17:**
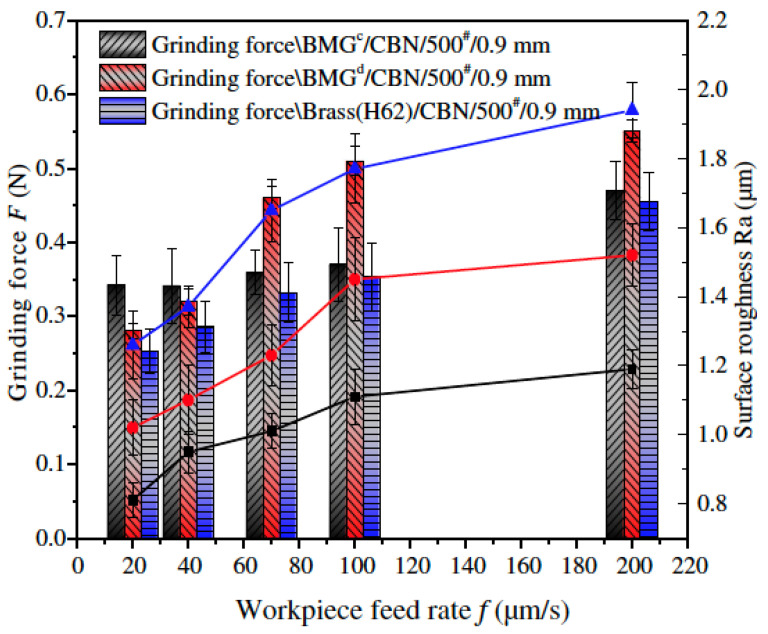
Variation of grinding force and average surface roughness with workpiece feed rate during micro-grinding of different BMG workpiece materials; The symbol # indicates grain size number of the grinding wheels [88]. (With kind permission from Springer).

Yin et al. (2017) [88] investigated grinding force ratio, surface roughness, and the material removal mechanism involved in micro-grinding of Zr-based metallic glass (BMG^C^ Zr_70_Ni_10_Cu_20_)_82_Ta_8_Al_10_ and BMG^D^ Zr_55_Cu_30_Ni_5_Al_10_). They reported a decreased value of micro-grinding force with increased spindle speed. Decreased force resulted in an eventual decreased roughness of the ground surface. Increased spindle speed caused an increase in the total abrasive number per unit time, which eventually reduced grinding force. In addition, an increased total abrasive number resulted in finely divided grinding chips as well as increased abrasive involvement in sliding and plowing. Therefore, the frequency of engagement for a single abrasive grain over a specified cutting area increased and the surface roughness was reduced. The increase in depth of cut also increased grinding force. Surface roughness, even though it exhibited a decreasing trend initially (2–6 μm), eventually increased in the range of 6 to 10 μm depth of cut. Undeformed chip thickness as well as contact arc length of grinding tool increased with the increase of depth of cut, which eventually resulted in an increased number of grains in contact, thus increasing grinding force. In addition, increased feed rate increased the micro-grinding force and roughness due to the increase in arithmetic deviation of surface contour, which occurred due to the increase in uncut chip thickness and reduced contact points between the abrasives of grinding tool and workpiece. An empirical model based on the simple single factor test, as proposed by this study, demonstrated optimum grinding parameters (grinding depth of cut of 6 μm, spindle speed of 60,000 rpm, and the workpiece feed rate of 20 μm) for achieving the best surface quality in BMG. A grindability study of BMG^c^, BMG^d^, and conventional polycrystalline metallic (brass H62) reported the highest micro-grinding force for BMG^c^ and the lowest force for the brass H62, as shown in Figure 16 [88]. This is due to the fact that brass H62 has the lowest hardness as well as better plasticity both in hot and cold states. It is also evident from Figure 17 that roughness experienced by brass H62 is higher than other BMGs due to the plastic and brittle mode of material removal. Nevertheless, both BMG^c^ and BMG^d^ experienced a plastic mode of material removal.

**Figure 18:**
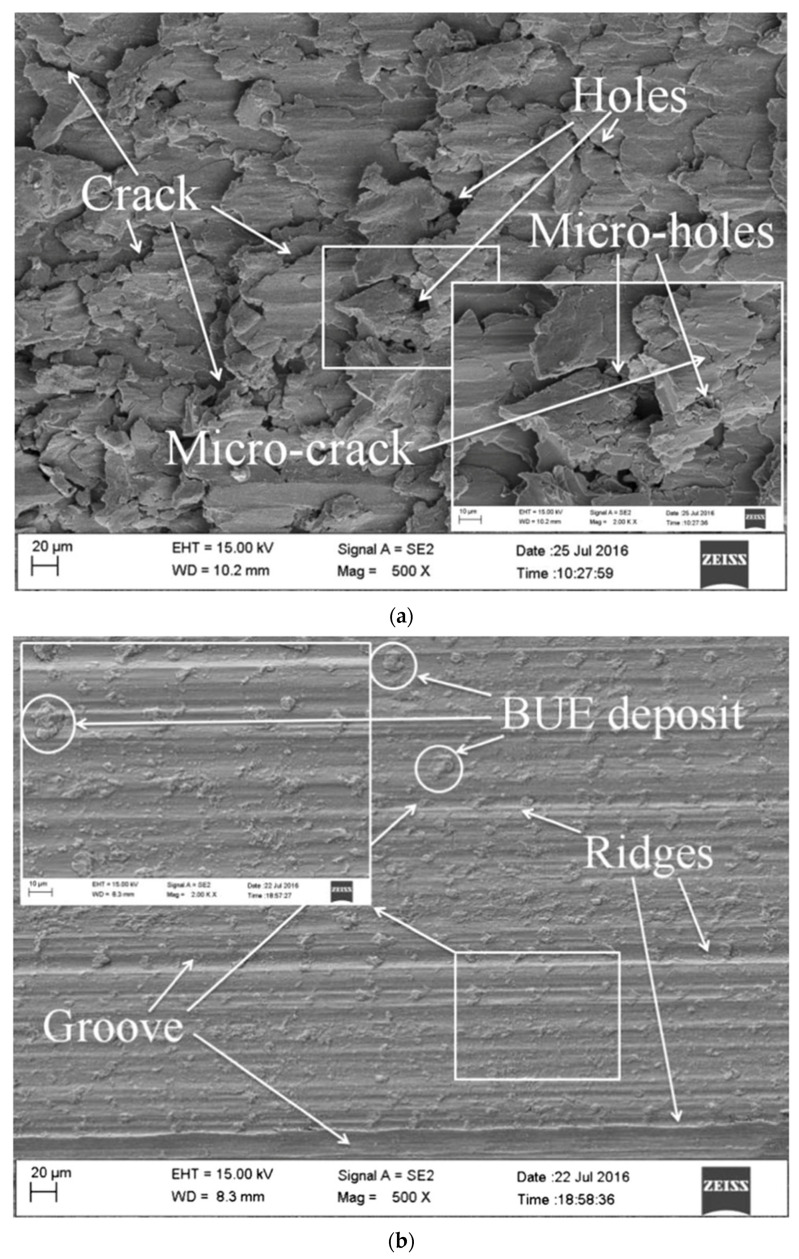
Images of grinding surface on BMGc generated by the (**a**) diamond and (**b**) CBN wheel [88]. (With kind permission from Springer).

Ya-Dong et al. (2018) [89] investigated the grinding performance of Zr-based BMG using CBN and diamond wheels. A comparative study using a CBN and diamond wheel revealed that the material removal rate differed from CBN to the diamond tool. Despite their unsuitability to react with Fe or other elements at high temperatures, chemical inertness and thermal stability make the CBN tool particularly suitable for grinding BMG. As can be seen from Figure 18, the surface generated by the CBN tool exhibited built up edges, ridges, and flow grooves [89]. On the other hand, the surface generated by the diamond tool exhibited micro cracks and micro-holes as a consequence of the lamination effect of BMG, without any sign of plastic grooves. The surface generated by the diamond tool possessed isotropic waviness whereas that by CBN possessed anisotropic waviness. In addition, the abrasive size and grinding tool diameter influenced the grinding output. An abrasive grain size of 500# experienced more grinding force than a grain size of 200#. A large diameter abrasive produced relatively less friction than a smaller one and consequently generated less heat, which caused increased grinding temperature as well as grinding force due to easy blockage on the grinding tool. Moreover, the grinding force produced by the larger diameter grinding wheel was higher than the smaller diameter wheel, as wheel diameter appeared to be in a linear relationship with grinding force. Increased wheel diameter also aided in increasing the linear velocity of the tool at any circumference.

In the study conducted by Lavisse et al. (2020), the microstructural changes were investigated during grinding of BMG [90]. Under 100 µm depth of cut, crystalline phases were noticeably found. It was also ensured that below 50 microns depth of cut, XRD was required to examine the possibility of formation of crystalline phases. Three various cutting conditions were revealed during this study, such as safe conditions, conditions that only affected the top BMG surface, and the condition which severely damaged the whole BMG piece. It was resolved that this study can be beneficial in the industrial sector while safely grinding BMGs. Table 1 shows the summary of the research carried out on conventional machining of BMG, the challenges reported, and solutions offered.

## 4. Non-Conventional Machining of BMG

### 4.1. Electro-Discharge Machining (EDM)

Electro-discharge machining (EDM) is a process that offers flexible machining of three-dimensional (3D) structures and functional surface generation in hard, brittle, and difficult-to-cut materials, as long as they are electrically conductive [91]. EDM works by inducing electrical sparks in the presence of dielectric fluid between an electrode and a work piece. There are several variations of EDM processes, however, there are two major variations: wire EDM and die-sinker EDM. In addition, the EDM has seen application in surface modification from macro to micro scale [92,93]. In die-sinker EDM, the electrode erodes as it encounters the workpiece to produce a mirror shape of the electrode on the workpiece [94]. Wire EDM uses an electrically conductive metal wire to follow a programmed path to machine 3D complex-shaped parts from an electrically conductive workpiece metal block [95]. Micro EDM differs from traditional EDM as micro EDM has a comparatively lower discharge energy level, smaller electrodes, and lower axis resolution. Micro EDM is now capable of machining complex 3D micro-features in a wide range of materials, from non-conductive [96] to semi-conductive materials [97], with high aspect ratios, such as micro-holes, micro-electrodes or shafts, micro-molds, etc., irrespective of the hardness of the workpiece. In recent years, the EDM process has also found application at nano scale for various applications such as nano-pores for DNA detection devices and nano-holes for next generation fuel injection nozzles [98]. However, given the excessively high temperature of the EDM process, unoptimized parameters may cause unexpected heating and cooling resulting in crystallization as well as oxidation of machined surfaces, and thus, changes in surface composition and microhardness [99].

**Figure 19:**
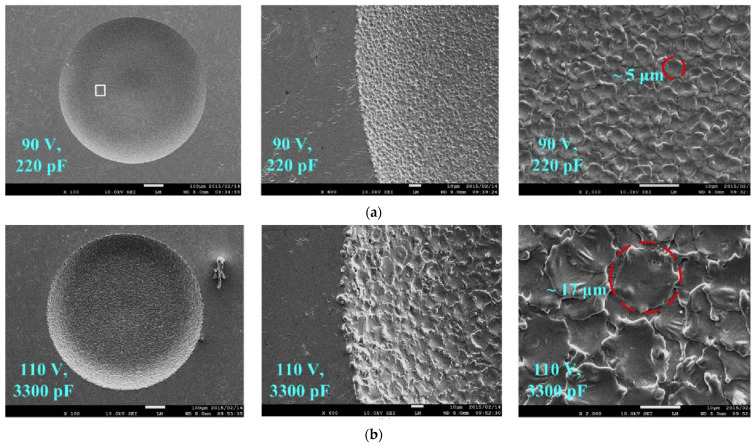
SEM images showing surface finish and craters of the micro EDM processed BMG surfaces at a (**a**) a lower setting of voltage and capacitance (voltage of 90 V and capacitance of 220 pF) and (**b**) a higher setting of voltage and capacitance (voltage of 110 V and capacitance of 3300 pF) [100]. (With kind permission from Elsevier).

**Figure 20:**
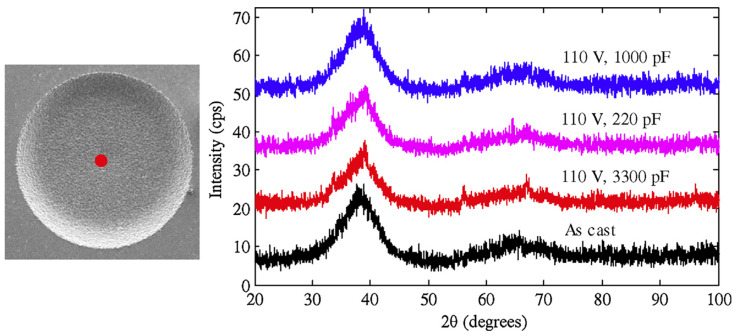
XRD patterns of the machined surface of BMG after micro EDM at 110 V and various capacitances; The ‘red dot’ in the left figure indicates the position where the XRD analysis was performed [100]. (With kind permission from Elsevier).

Huang and Yan (2015) [100] investigated the machinability of Zr-based BMG using micro EDM. They investigated surface morphology, surface roughness, crystallization, material removal rate, and carbonization during micro EDM of BMG using a copper electrode. Through the experiments, the two major parameters of discharge voltage and capacitances were varied, whereas the depth of cut, feed rate, and rotational speed was constant throughout the process. It was found that the material removal rate increased with an increase in capacitance and voltage. This was found to be due to the discharge energy increasing with the increase in capacitance and voltage. A tapered shape was obtained with decreasing voltage as the wear of the electrode became more significant. The surface roughness decreased with decreasing capacitance at the same voltage. Surface roughness also reduced with decreasing voltage because of the decreased crater size. Carbonization was seen at higher capacitances during micro EDM of BMG. Interestingly, the carbons that were deposited also had amorphous structure. However, the combination of carbon and Zr lead to partial crystallization on the machined surface of BMG. There was no impact on the intensity of crystallization. Figure 19 shows the machined surface morphologies of BMG obtained at different settings of voltages [100]. The XRD patterns of the machined surface, as shown in Figure 20, show the presence of ZrC on the machined surface [96]. As there was no or minimum presence of ZrO_2_ on the machined surface, there was minimum crystallization on the machined surface. This is opposite to the characteristics shown by the conventional EDM machined surface. This may be due the difference in level of discharge energy used in conventional EDM and micro EDM. It was concluded that micro EDM retained the amorphous structure of BMG more than conventional EDM, mainly due to the significantly lower discharge energy used compared to conventional macro scale EDM.

In another research, Huang and Yan (2016) [101] studied micro EDM of Zr-based BMG and performed grinding operations after the micro EDM using a polycrystalline diamond tool. Since metallic glass can go through failure because of its confined tensile plasticity, grinding was suggested for removal of crystallized surface. In this study, an electrode rotational speed of 3000 rpm, two voltages, 70 V and 110 V, and two capacitances, 3300 pF and 1000 pF, were used, while keeping the depth of cut in Z direction constant. Carbon-free electrodes caused crystallization due to Zr reacting with carbon to form zirconia carbide (ZrC). For the same capacitance, a decrease in voltage resulted in an increase in surface roughness because of micro-voids. For the same voltage, a decrease in capacitance reduced the surface roughness. Decrease in crater size could be the cause of better surface finish. Surface finish was further improved after grinding. Grinding with a different depth of cut showed that the surface had hierarchical crystallization along the depth direction. Overall, using micro EDM followed by grinding can be an effective method to remove carbonized and crystallized surface. Almost all of the crystallized part can be removed when grinding is done after micro EDM up to a 20 μm or 30 μm depth, as the XRD spectrums of these two cases are very similar to that of as-cast BMG.

Chen et al. (2008) [102] explored influence of micro EDM on multiple bulk metallic glass (A: La-Al-Ni-Cu, B: Zr-Al-Ni-Cu, C: Cu-Zr-Al-Y) alloys to examine surface morphology and burr formation. It was found that BMG material C produced the smallest crater size and heat-affected zone when the machining was performed with micro EDM. The structure remained the same for all BMG materials during micro EDM. It was also found that the heat-affected zone was the highest in BMG A because of its low thermal conductivity. All three alloys showed no devitrification after micro EDM. Lower electrode diameter resulted in greater deterioration of the machined hole as the diffusion of heat became difficult with a smaller diameter. It was corroborated that use of micro EDM did not affect the amorphous nature of the chosen metallic glass.

**Figure 21:**
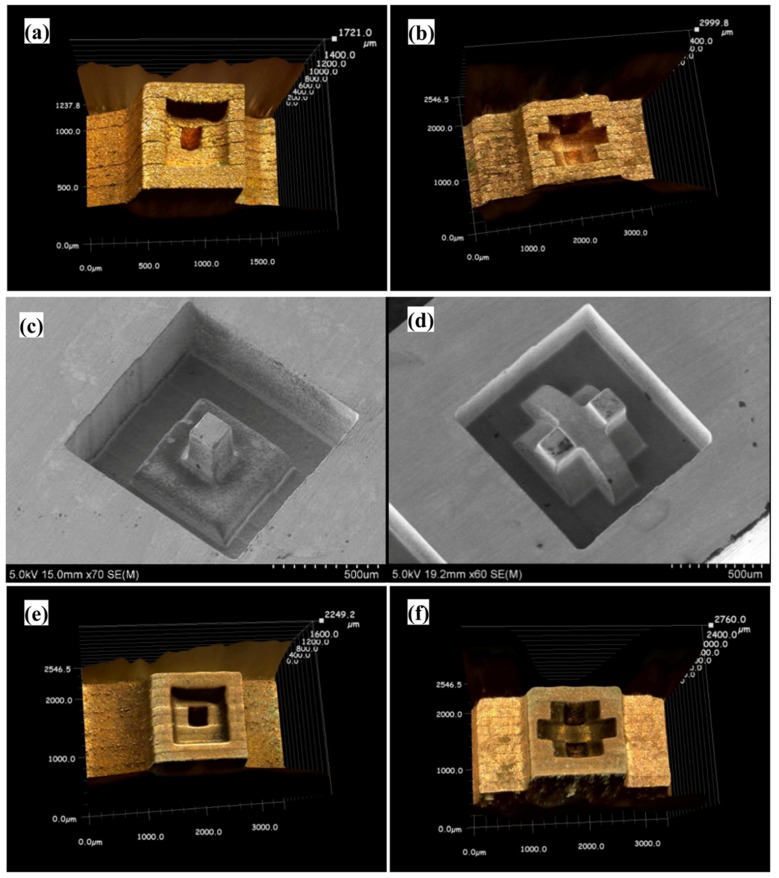
(**a**,**b**) Three-dimensional images of micro-electrode and micro-mold; (**c**,**d**) SEM images of three-dimensional micro-molds machined by the micro EDM process; (**e**,**f**) micro-electrodes after used in micro EDM [103]. (With kind permission from IOPScience).

Xu et al. (2016) [103] took this investigation further by using 3D manufactured electrodes to fabricate micro-molds in Pd-Cu-P-Ni metallic glass using micro EDM. Throughout the experiment, a pulse frequency of 0.2 MHz, pulse width of 800 ns, pulse interval of 4200 ns, and voltage of 80 V were used. Figure 21 shows the fabricated micro-molds in BMG using the micro EDM process [103]. Copper foil was used to develop the electrode for micro-machining. The dimensional accuracy provided by the electrode was within 8 μm. This ensured dimensional precision while cutting. Processing depths were varied, and it was concluded that the increase in depth resulted in shallow craters. This can be attributed to a better surface on the electrode itself. On the first use, electrode had a rough surface and the increase in depth improved the surface of the electrode, which resulted in a better surface of the micro-mold. Carbon deposition, however, increased as the processing depth increased. It was found that Cu and P phase were formed on the machined surface, resulting in the crystallization of the BMG surface after the machining process.

**Figure 22:**
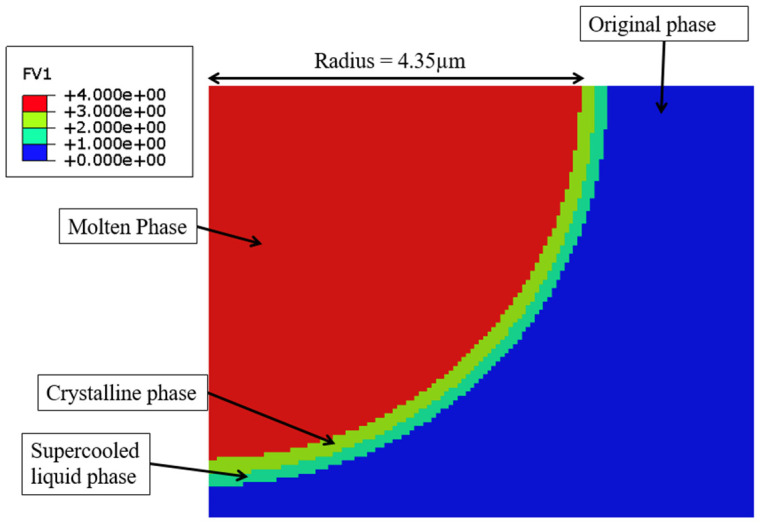
Phase distribution for a typical crater generated during micro EDM of BMG showing crystalline layer followed by supercooled liquid phase. The crater was generated using a discharge energy of 5.5125 μJ (parameter setting: voltage of 105 V and capacitance of 1000 pF) [104]. (Copyright: Elsevier, Open Access).

Liu et al. (2019a) [104] carried out an experimental investigation and numerical simulation on the machining behavior of ‘Vitreloy 1’ (Zr_67_Cu_10.6_Ni_9.8_Ti_8.8_Be_3.8_ (wt%)) BMG by micro EDM. In the experimental investigation, the influence of micro EDM parameters, i.e., voltage and capacitance, on the crater size, recast layer, and overcut of the machined features was investigated. It was reported that both the crater size and recast layer around the micro-holes could be reduced by minimizing the discharge energy per pulse. A crater size as small as about 2 microns could be obtained by reducing the discharge energy level and by the reduction of gap voltage and capacitance. The study also developed a mathematical model to simulate crater formation during micro EDM of BMG. It was found that the crater formation in BMG was associated with a crystallized region, followed by a supercooled liquid phase, which together formulated the recast layer around the crater. The amount of crystallization increased with the increase of gap voltage and capacitance due to an increase in discharge energy. Figure 22 shows phase distribution of a crater formed during micro EDM of BMG using a discharge energy of 5.5125 μJ that was generated using a discharge voltage of 105 V, and a capacitance of 1000 pF [104].

**Figure 23:**
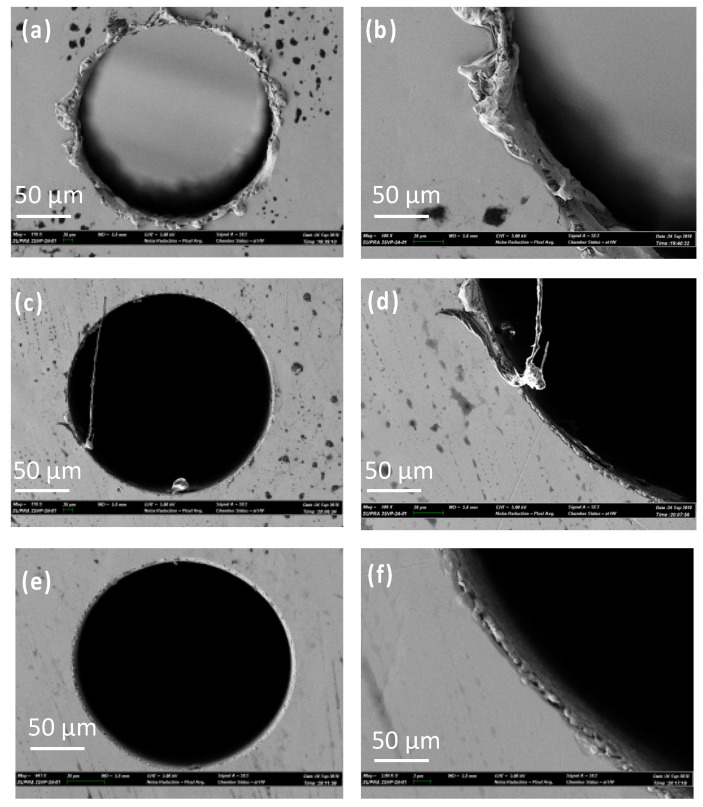
Comparison of surface quality and recast layer around the micro-holes machined at (**a**,**b**) 400 nF, (**c**,**d**) 1 nF, and (**e**,**f**) 0.01 nF at 80 V [105]. (Copyright: Elsevier, Open Access).

In another study, Liu et al. (2019b) [105] investigated the micro EDM machinability of ‘Vitreloy 1’ BMG, with a goal of achieving high-aspect-ratio micro-holes using micro EDM drilling. They investigated the effects of electrical and non-electrical parameters on the high-aspect-ratio micro EDM drilling capability in BMG in terms of overcut and taper angle of fabricated micro-features. They found that, among all the parameters, capacitance had the most significant effect in determining surface quality and recast layer around the micro-holes. With increased capacitance, the surface became rougher, and the thickness of the recast layer increased. Gap voltage was found to have more of an effect on the overcut of the micro-holes with increasing overcut and increased gap voltage. Higher electrode rotational speed was found to increase MRR and reduce tool wear due to improved flushing conditions, whereas lower settings of electrode rotation speed were found to provide improved dimensional accuracy by providing lower overcut and taper angle. It was also reported that the amount of migration of materials was reduced by reducing the discharge energy level. Figure 23 shows the reduction in recast layer and migration of materials to the edge of the micro-hole by reducing the values of capacitance, while keeping all other parameters constant [105]. It can be seen that by the reduction of capacitance, i.e., discharge energy, the recast layer can be significantly minimized. This is because when using an RC-type pulse generator, as was used in this study, the discharge energy can be minimized to a great extent. In addition, the RC-type pulse generator is known to generate nanosecond pulse duration compared to the typical micro-second pulse duration generated in transistor-type pulse generators [106].

In a separate study, micro-holes were fabricated on Zr-based metallic glasses using micro EDM to investigate drilling precision, where tungsten material was used as the tool electrode [107]. The research revealed that higher voltage and reduced capacitance, along with a non-rotating tool, stabilized EDM drilling, resulting in minimal edge deviation and taper angle. Tool rotation had a significant impact on overcut, edge deviation, and taper angle, but it did decrease machining time. Conversely, lower voltage, moderate capacitance, and a non-rotating tool influenced machining by reducing overcut, specifically to 0.75 µm at the entry and 0.5 µm at the exit. The researchers concluded that tool rotational speed played a crucial role in enhancing hole accuracy and reducing machining time.

The performance of wire EDM in machining Zr-based BMG was assessed by Chang et al. [108] They further compared the wire EDM machinability of BMG with that of heat-treated BMGs (HBMGs) and industry-grade pure zirconium [108]. Molybdenum wire was used as the tool electrode during wire EDM, whereas a mixture of water and base oil (water: oil = 19:1) was considered as the dielectric medium. Six processing parameters, such as pulse type (GP), pulse on-time (ON), pulse off-time (OFF), peak current (IP), servo voltage (SV), and the running frequency of the wire cylinder (WS) were varied in order to observe the effects of these parameters on the cutting efficiency (CE) and surface roughness (Ra). The average surface roughness (Ra) decreased, whereas CE increased as the pulse type was altered from rectangular to packet pulse. A lower frequency was served by the packet pulse, which was attributed to the lower values of CE. On the other hand, higher energy per unit time was generated during the rectangular pulse, which resulted in a higher temperature at the time of sparking. The higher temperature decreased the viscosity of the melted BMG and caused a stronger throw-out effect and eventually resulted in smoother craters and lower Ra values. It was found that CE values increased with a rise in pulse on-time (ON) and peak current (IP).

In a different study, Zirconium-based metallic glass underwent machining through multiple passes of a wire electrode in the wire EDM process [109]. Molybdenum wire with a 0.18 mm diameter was employed as the tool electrode, while a water-soluble cutting fluid served as the dielectric medium. The outcomes of this study indicated that, as the number of wire passes increased, both the size of the crater and the surface roughness decreased, leading to a smoother surface. Nevertheless, the microhardness values declined as the number of wire passes increased and, after the fourth pass, the micro-hardness value closely approximated to that of the base BMG material. Following the initial cut, the heat-affected zone displayed numerous microcracks, which were subsequently eliminated during further trimming processes. Ultimately, after the fourth cut, the thickness of the recast layer was reduced to 52%.

Tang et al. developed a simulation model utilizing the finite element method to explore the impact of pulse on-time and peak current on the surface roughness when machining Zirconium-based BMG using wire EDM [110]. They also conducted experimental research to validate the accuracy of the simulation model. The dielectric fluid employed was a mixture of water and base oil, with a ratio of 19:1. The simulation findings revealed that, as both pulse on-time and peak current increased, the spacing between the heat source points and the discharge energy both increased. This resulted in deeper erosion pits and a rougher surface. The experimental data corroborated these simulation results, although the average relative errors were 2.53% and 2.87% for peak current and pulse on-time data, respectively. The authors concluded that by adjusting the parameters in accordance with the findings of the simulation study, it was possible to significantly control the surface topology and machining precision.

In another research study, Zr57 BMG was machined through sinking EDM while keeping the same tool electrode [111]. The results were compared to those obtained from crystallized BMG, Zr702 BMG, and TC4 alloys in terms of varying dielectric mediums and processing parameters. The Zr57 sample that underwent sinking EDM under suitable machining conditions exhibited superior surface finish in comparison to the samples machined through the wire EDM process. Additionally, it was found that using kerosene as a dielectric fluid resulted in a lower surface roughness when contrasted with EDM oil. However, a higher degree of carbonization occurred due to the decomposition of kerosene. Rapid cooling of melted alloys was effective in minimizing crystallization. During the machining of Zr57, the study observed losses of copper and nickel. The minimum thickness of the recast layer obtained was less than 9 µm, which contained an amorphous matrix and carbides. It was resolved that sinking EDM offered a practical solution for machining hard-to-cut BMGs without significant loss of the parent material, provided that appropriate machining parameters were used.

**Figure 24:**
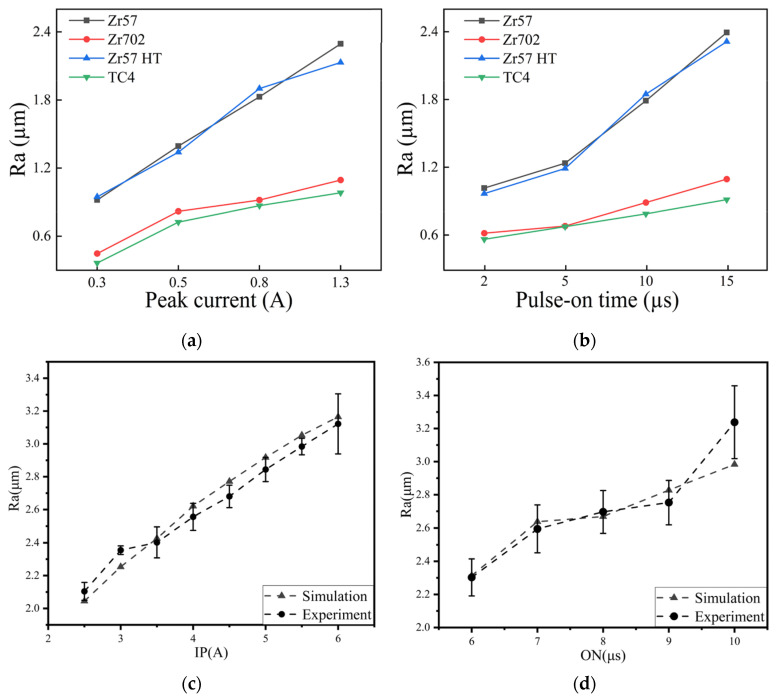
Comparison of the effects of peak current (IP) and pulse duration (ON) on the average surface roughness of the machined BMGs using (**a**,**b**) die-sinking EDM [111] and (**c**,**d**) wire EDM [110]. (With kind permissions from Springer).

The study related to [111] reported die-sinking EDM of bulk metallic glass where effects of peak current, pulse on time, and pulse off time on MRR and surface roughness were investigated. The study related to [110] reported on wire EDM of Zr57 BMG where effects of peak current was investigated on surface roughness. Although both are variations of EDM process, WEDM uses wire as a tool electrode and die-sinking EDM uses a cylindrical rod as electrode. In addition, their process parameters, such as peak current, pulse on and pulse off time, were varied within different ranges. However, both studies reported on one common conclusion: with the increase in peak current and pulse duration, surface roughness showed an increasing trend, as shown in the Figure 24 [110,111].

BMG composites (BMGCs) exhibit superior mechanical properties compared to BMGs, but machining BMGCs presents a significant challenge due to their difficult-to-cut nature. Chen et al. employed the die-sinking EDM process to machine BMGCs and conducted a comparative analysis with that of commercially available pure Zr702, considering both rough and refined conditions with respect to material removal rate, surface roughness, and elemental variation [112]. Copper metal served as the tool electrode, and EDM oil was utilized as the dielectric medium. The study revealed that the peak current played a pivotal role in determining material removal rate and surface roughness, with pulse on-time following closely behind. In contrast, gap voltage and pulse off-time had limited influence. BMGCs subjected to the EDM process exhibited superior surface quality when compared to Zr702. Additionally, the researchers observed carbonization occurring on ridges rather than at the bottom of the craters, producing varying outcomes under different conditions.

### 4.2. Electrochemical Machining of Bulk Metallic Glass

Electrochemical machining (ECM) is a process of machining electrically conductive materials through electro-chemical dissolution. The chemical dissolution happens by using a cathode and an electrode with electrolyte and an anode work piece, and bringing the anode closer, within few microns, of cathode. Micro ECM produces microstructures with fine surface finish and with minimum crystallization. However, due to chemical exposure, machining of BMG with micro ECM is a challenge, as metallic glasses have high chemical resistance. Several factors need to be controlled during machining using micro ECM, such as electrolyte, electrodes, distance between workpiece and electrode, and other pulse parameters such as duty cycle, voltage range, and peak voltage. There have been several research studies on the micro ECM of BMGs.

**Figure 25:**
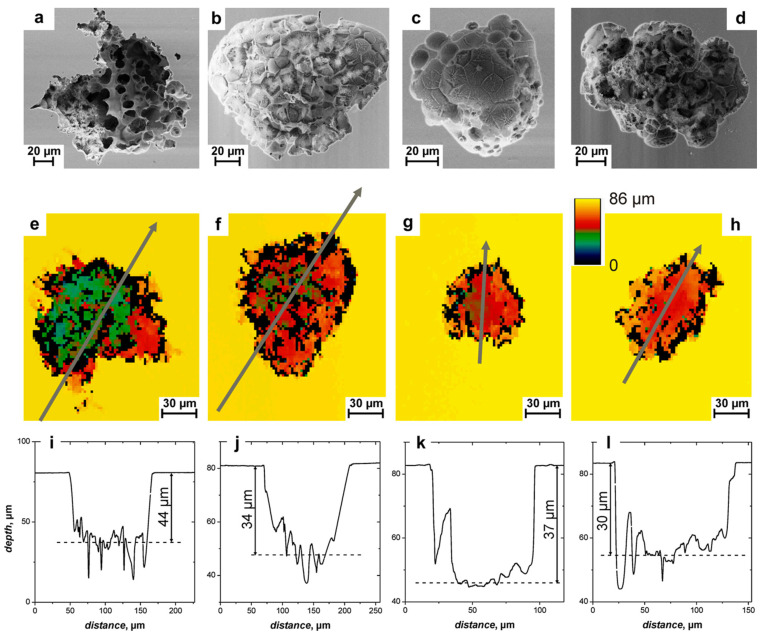
SEM images (**a**–**d**), corresponding depth profiles (**e**–**h**), measurements (**i**,**j**) of machined structures on the Zr BMG at different voltage levels: 4 V (**a**,**e**,**i**), 5 V (**b**,**f**,**j**), 6 V (**c**,**g**,**k**), and 7 V (**d**,**h**,**l**). t_on_ = 100 ns; A3 electrolyte [113]. (With kind permission from Elsevier).

Koza et al. (2011) [113] introduced electrochemical micromachining on Zr-based bulk metallic glass by varying pulse voltage and pulse length. A micro tool electrode was used in their experiments to fabricate micro-holes on the BMG surfaces. Some of the challenges that were seen were choice of appropriate electrode, distance of electrode from the workpiece surface, pulse parameters, and choice of electrolytes. The authors utilized a WC-Co electrode, NaNO_3_, and A3 electrolyte. NaNO_3_ was found to be an unsuitable electrolyte for machining BMG due to the formation of corrosive layers on the surface of the machined parts. A3 electrolyte turned out to be a better option for machining BMGs. It was found that an increase in pulse voltage resulted in a smoothing effect, while short pulse length had a higher machining resolution. Figure 25 shows the SEM images and respective depth profiles of the machined micro features on BMG by micro ECM [113]. It was found that the machining of BMG using micro ECM was possible if the proper parameters and electrolyte were selected. The experiments revealed that controlling fast transpassive dissolution process, optimizing electrode distance, selection of optimum electrolytes and pulse parameters enhanced the machining process for micro ECM of BMGs.

**Figure 26:**
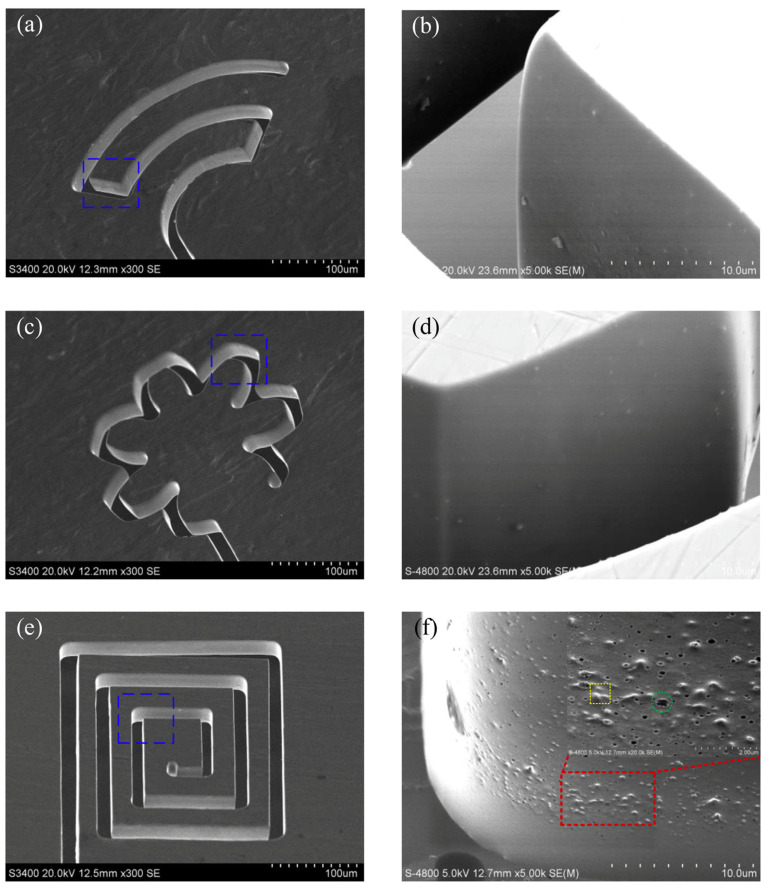
SEM images of microfeatures obtained in Ni-based BMGs: (**a**) cantilever beam, (**c**) micro gear, and (**e**) micro square helix. The magnified view of the surface of those features, as shown in blue and red color, is shown in (**b**,**d**,**f**), respectively [114]. (With kind permission from Elsevier).

Meng et al. (2017a) [114] conducted electrochemical machining on Ni-based metallic glass to study the impact of electrolyte and voltage pulse waveform on the quality of various microstructures like cantilever beam, micro gear, and micro square helix parts. It was found that tool wear and heat induction were not significant, and the material removal rate was not affected by the hardness of the BMG. Tungsten wire was used as an electrode and chloric acid (HClO_4_) and sulfuric acid (H_2_SO_4_) were used as electrolytes. It was reported that slid electrolytes affected the electrical conductivity of the workpiece by filling the machined gap. NaNO_3_ produced a lower aspect ratio compared to H_2_SO_4_. Surface finish was found to be desirable in electrolytes of a high concentration. Uniform surface finish with passive film growth was seen on microstructures produced in sulfuric acid. Efficiency and stability of machining was improved with a voltage pulse rise time of 2 ns and methanol solvent in electrolyte improved the machining of BMG with a pulse-off voltage of 0.6 V. Figure 26 shows the micro features on Ni-based BMG workpiece machined by the micro ECM process [114].

In another study, Meng et al. (2017b) [115] explored micro patterning of Ni-based metallic glass using micro ECM. They investigated effects of parameters such as electrolyte concentration, voltage, pulse duration, pulse period, and feed rate on the machined slit width. Hydrochloric acid was used as an electrolyte. In order to reduce formation of bubbles, a travelling technique with cathode wire and vibration with anode were implemented. It was found that an increase in feed rate reduced the machined gap. In addition, short pulse duration with a low feed rate helped in producing a smaller side gap. A better surface finish was achieved with a steady machining condition with high feed rate. Current corrosion was found to be reduced as the secondary processing time decreased. It was seen that the reduction in applied voltage also reduced the width of the gap. The primary reason for this observation was the reduction in the machining gap and limited movement of the electrolyte. When the pulse duration was held constant, increasing the pulse period provided better accuracy for the machined features. A low concentration of electrolyte produced a better slit width and the optimal HCl concentration for micro ECM of BMG was proposed to be 0.1 M.

**Figure 27:**
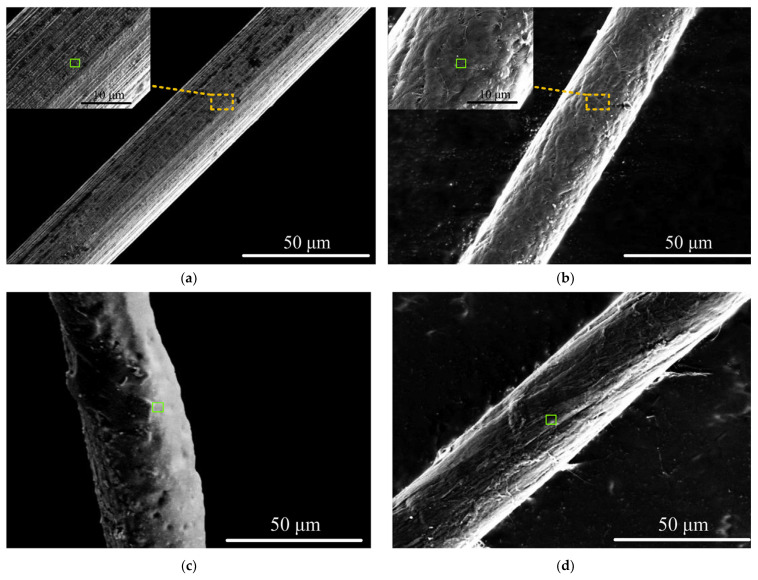
SEM images showing tungsten wire and CNF before and after use in micro ECM: (**a**) tungsten wire before ECM, (**b**) CNF before ECM, (**c**) tungsten wire after being used as electrode in ECM, (**d**) CNF after ECM; the colored boxes indicates from where magnified views were taken [116]. (With kind permission from Elsevier).

Carbon nanotubes (CNT) are a very strong, flexible yet lightweight material that can carry high currents. Meng et al. (2017c) [116] experimented with carbon nanotube fiber (CNF) as an electrode to machine nickel-based bulk metallic glass. With proper mass transport, machining can be improved to obtain better quality machined parts and attain better stability while machining. Flow field simulation and hydrophilicity analyses were performed in this study to investigate the efficiency of the electrode during machining. Mass transport with the use of CNF was improved as the electrolyte was destabilized quickly and the irregular motion of electrolysis was accelerated. Electrical conductivity was improved in carbon nanotubes by electroplating with an Ni layer. Tungsten and CNF electrodes before and after being used in the ECM process are shown in Figure 27 [116]. Compared to the tungsten electrode, CNF had greater surface of hydrophilicity in both sulfuric acid and hydrochloric acid. Viscosity increased with hydrophilicity, which then promoted mass transport. The feed rate also improved with the use of the CNF electrode. A smaller pulse duration with maximum tension can result in a narrower slit during machining. A stable passive film was formed with use of sulfuric acid, which in turn improved surface finish and localization. The passive film had a less protective effect as the current density increased, which also increased passivation potential. Overall, CNF was found to be a great electrode in electrochemical machining of nickel-based bulk metallic glass.

Cole et al. (2017) [117] studied the influence of micro ECM parameters on the machined surface of Zr-based bulk metallic glass. The micro tool electrode caused little to no crystallization on the machined BMG surface. Copper with silver paint was used as an electrode during the experiment, whereas NaNO_3_ was used as an electrolyte. Re-passivation was prevented using a voltage of 2.235 V during the machining process. Duty cycle, voltage range, and electrolyte flow rate were changed to observe their effects on machining performance. The Taguchi method was used for analyzing the effects of machining parameters on the machining performance. Due to corrosiveness of Zr BMG, parameters were carefully chosen. The experiments determined that the use of a 1:10 duty cycle and 0.4 L/min flow rate of electrolyte produced a shape with good tolerance and surface finish. Overall, aqueous solution electrolyte, shorter pulse times, and slower movement speeds were recommended for machining complex shapes.

**Figure 28:**
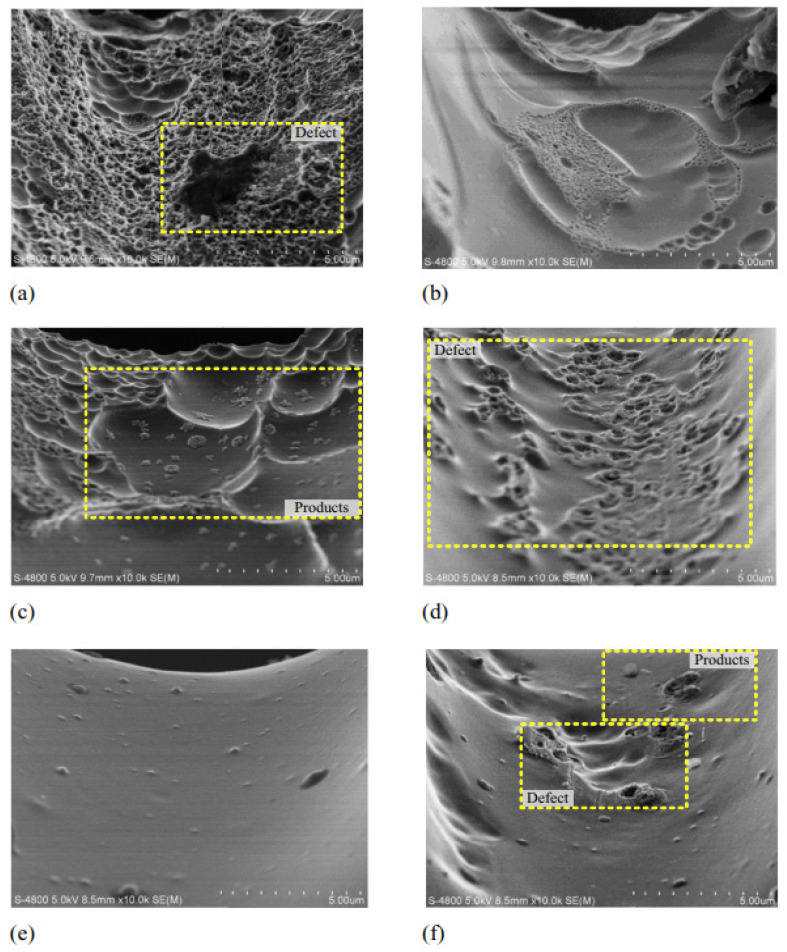
SEM images of BMG surfaces after ECM using various concentrations: (**a**) 0.05 M, (**b**) 0.10 M, (**c**) 0.50 M in HCl, and (**d**) 0.05 M, (**e**) 0.10 M, and (**f**) 0.5 M in H_2_O_4_ [118]. (With kind permission from IOPScience).

Zeng et al. (2017) [118] conducted ECM of Ni-based metallic glass to investigate surface characteristics after machining. Current density, mass transport, and passivation were used to examine the surface finish of the specimen. The machined surface was found to be smoother when sulfuric acid in 0.1 M concentration was used. This concentration of the electrolyte was found to promote passivation. Figure 28 shows the images of the machined surface under different concentrations [118]. Furthermore, the effect of electrolyte polishing on the surface was observed as the process of machining continued. In contrast, HCl at 0.1 M concentration provided comparatively higher surface roughness on the parts. Use of a moving cathode and anode supported mass transport during the ECM of BMG. Moreover, higher current density enhanced surface morphology as the voltage and feed rate was larger with a comparatively higher current.

Sueptitz et al. (2015) [119] carried out electrochemical machining of Fe-based BMG to study the transpassive dissolution regime. Their experiments discussed ways of machining BMG under acidic aqueous electrolyte using electrodes made from tungsten (W) and platinum (Pt). Stainless steel was also machined as a basis of comparison with the performance of BMG. It was found that low pulse-off voltages resulted in no material removal. High pulse-on and pulse-off voltages resulted in irregular structures. From the study it was concluded that sulfuric acid with an addition of Fe_3_(SO_4_)_3_ reduced evolution of hydrogen, which was achieved by reducing the charge transfer at the electrode. In addition, combination of Schottky diode and Fe_3_(SO_4_)_3_ in electrochemical machining produced a better surface finish on the BMG.

### 4.3. Ultrasonic Machining of Bulk Metallic Glass

Ultrasonic machining uses ultrasonic vibration (greater than about 20 kilohertz) to remove materials by the process of cavitation as the primary material removal mechanism and secondary abrasive action. Ultrasonic machining uses a metallic tool and abrasive slurry solution at the gap between the tool and the workpiece and ultrasonic vibration of the metallic tool to remove material from the workpiece in the form of craters or chipping. This form of machining is very effective for machining brittle and difficult-to-cut materials with high aspect ratio structures. Very few research studies have been conducted using USM for machining bulk metallic glasses.

**Figure 29:**
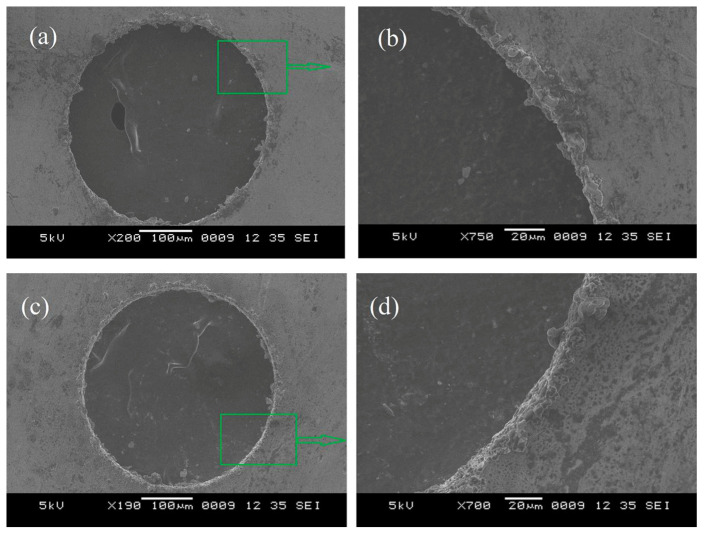
SEM images of micro-holes on Zr-Cu-Ti metallic glass using a stainless-steel tool under the presence of boron carbide abrasive slurry; (**a**) entry side of the hole, (**b**) magnified image of entry hole edge, (**c**) exit side of the hole, and (**d**) magnified image of exit hole edge [120]. (With kind permission from Elsevier).

Kuriakosea et al. (2017) [120] explored ultrasonic machining on ZrCuTi metallic glass using a hollow stainless-steel tool as a tool head. During the experiments, machinability of bulk metallic glass was studied using overcut, edge deviation, taper angle, tool wear rate, and material removal rate. Boron carbide (B_4_C) was used as an abrasive slurry, where abrasive grit sizes of 400 and 600 were used. It was seen that overcut was decreasing with an increase in feed rate. Abrasive of concentration 50–60% in the slurry yielded better results, and overcut was lesser for lower grit size. Edge deviation was found to be better with higher abrasive sizes, and lower deviation was obtained with moderate feed rate and slurry concentration. Lower slurry concentration meant that flow of slurry into the machining zone was slower. Lower slurry rate and higher feed rate tended to have an increased material removal rate. A tapered angle was found to be higher for the drilled micro-holes with higher grits. As shown in Figure 29 [120], micro-holes obtained using USM of BMGs do not have very clear edges because of the chipping nature of the material removal rate in USM. However, with careful selection of parameters, grit sizes, abrasive particle, and slurry liquid and concentration, micro-holes with acceptable rim edges and accuracy can be machined on BMGs using the USM technique.

### 4.4. Laser Machining of Bulk Metallic Glasses

Laser machining uses a controlled laser beam to heat and remove materials from the target workpiece. Melting and evaporation are the major material removal mechanisms in laser machining where no physical tool is used, as it occurs in conventional machining. Laser machining is another technique used for machining difficult-to-cut materials, as it is capable of machining materials irrespective of their hardness. As a result, laser machining has been tried by several researchers as a method for machining BMGs. However, the major challenge in laser machining of BMGs is the high degree of crystallization at the surface, sub-surface, and around the machined features due to the high temperature generated during machining. Therefore, one of the major research questions that is explored by researchers is how to minimize the crystallization during laser machining of BMGs.

**Figure 30:**
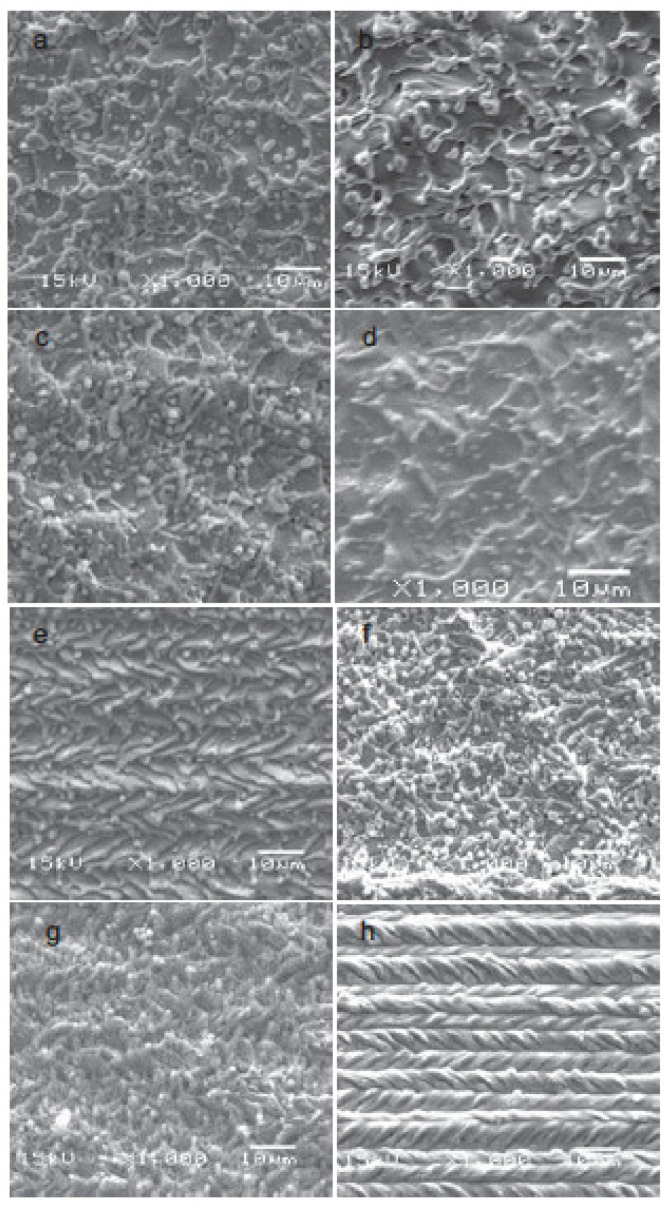
SEM images of machined surfaces of BMGs after being processed by laser at various parameter settings; (**a**–**h**) indicates different settings of parameters with varied parameters, (**a**) 1.77 W, (**b**) 2.13 W, (**c**) 0.16 W, (**d**) 3.01 W, (**e**) 2.62 W, (**f**) 5.30 W, (**g**) 0.25 W, and (**h**) 1.96 W [121]. (Open access article).

Pavey et al. (2013) [121] studied Zr-based bulk metallic glass under a nanosecond laser. It was found that crystallization did not occur with laser machining, which is very surprising considering the peak temperature generated during laser machining. In the research, effects of various laser parameters such as power, repetition rate, pulse energy, track distance, peak power, layer thickness, and fluence were investigated. It was concluded that the nanosecond laser preserves the mechanical properties of Zr BMG. Thermal diffusion reduction led to better morphology, and this can be obtained by using low peak power, low pulse frequency, and higher track distance. The surface roughness generated in nanosecond laser machining of BMG was on par with the surface roughness generated in micro milling. It was recommended to post-treat the specimen by annealing or in situ laser remelting before machining. It can be seen that the changes in XRD spectrum are at a minimum except for the fourth case, wherein additional peaks were observed due to crystallization on the machined surface. The SEM images of the laser machined surfaces are shown in Figure 30 [121], which demonstrates clear evidence of melting and evaporation on the machined surface.

Williams et al. (2016) [122] carried out a theoretical and experimental investigation on the formability of Zr-based bulk metallic glass using nanosecond laser processing. They proposed a theoretical model for laser material interaction for single pulse and carried out an experimental investigation under single laser pulses for validation. It was reported that pulses shorter than 25 ns produced comparatively smaller and cleaner craters, thus producing a smaller melt pool and a smaller heat-affected recrystallization zone at the machined sub-surface.

**Figure 31:**
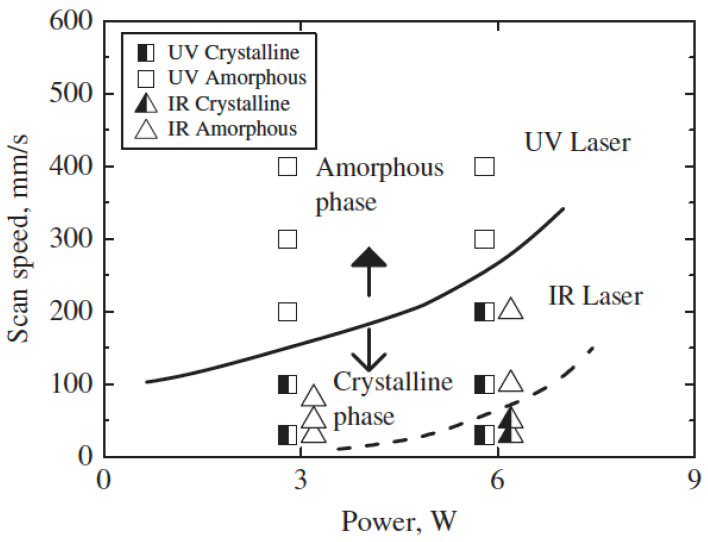
Scan speed vs. laser power graphs showing amorphous to crystalline phase transition of BMGs after laser processing [123]. (With kind permission from Elsevier).

**Figure 32:**
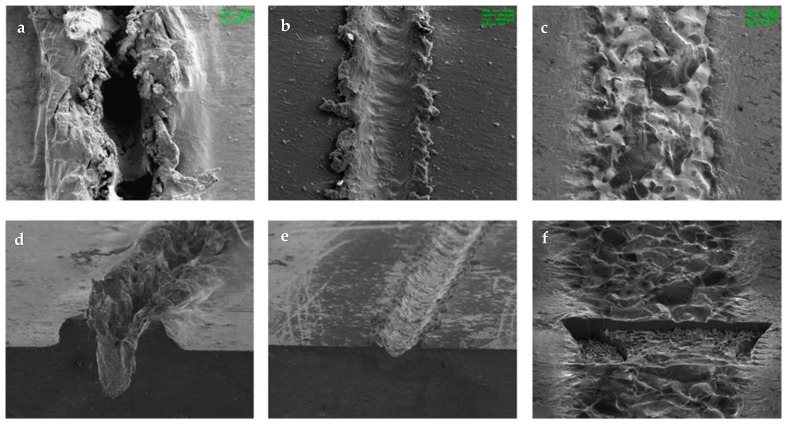
SEM images of machined features and respective surfaces of BMGs after laser micromachining: (**a**–**c**) top views of features, and (**d**–**f**) cross-sectional views; (**a**,**d**) UV laser, fluence of 12 J/cm^2^, scan speed of 30 mm/s, (**b**,**e**) UV laser, fluence of 12 J/cm^2^, scan speed of 300 mm/s, (**c**,**f**) IR laser, fluence of 19 J/cm^2^, scan speed of 30 mm/s [123]. (With kind permission from Elsevier).

Lin et al. (2012) [123] carried out micromachining of Mg-Cu-Gd bulk metallic glass using a 355 nm ultraviolet laser and 1064 nm infrared laser. Short pulses were used for machining to maintain the amorphous characteristics of the metallic glass. During the study, laser fluence and scan speed were varied. For a UV laser, it was found that an increase in scan speed and laser power decreased the desired cutting depth. Figure 31 summarizes cases wherein laser machining of BMG produces crystalline or amorphous phases with UV and IR [123]. Slow scan speed seemed to induce plastic wrinkles caused by the UV laser irradiation. It was seen that plastic deformation occurred due to thermal expansion. However, as the scan speed increased, thermal dissipation became faster, and wrinkles decreased. It was also observed that the amorphous structure turned into a crystalline structure when the laser fluence was increased and the scanning speed was increased. Figure 32 shows machined surfaces of micro-features under different processing parameters of BMG. Crystallization formed as a result of high power and low scan speed [123]. The UV laser had a higher absorption of irradiation than IR, as long wavelength has lower photon energy. Overall, micromachining with a UV laser produced an optimal result, and a fluence of 12 J/cm^3^ and scan speed of 300 mm/s were used.

Huang and Yan (2018) [124] reviewed the patterning process of BMG using laser surface processing. They reported various laser surface structuring mechanisms for BMG and their applications in functional surface modification. It was reported that laser surface irradiation had the capability of producing textured surfaces on BMG for potential applications in biomedical, waterproof, and catalysis areas. They also reported and discussed some distinguished characteristics of laser-processed BMG surfaces, such as ripple formation, formation of porous nanostructures on the machined surface, and line laser irradiation.

Jiao et al. (2019) [125] investigated the micro scale machinability of Vitreloy 105 (Zr_52.8_Cu_17.6_Ni_14.8_Al_9.9_Ti_4.9_) bulk metallic glass using a nanosecond laser. Their study had considered both theoretical and experimental aspects to study the effects of laser parameters on the laser micromachining performance of BMG. The authors also modeled how the single pulse and multiple laser pulses interacted with the bulk metallic glass during micromachining.

**Figure 33:**
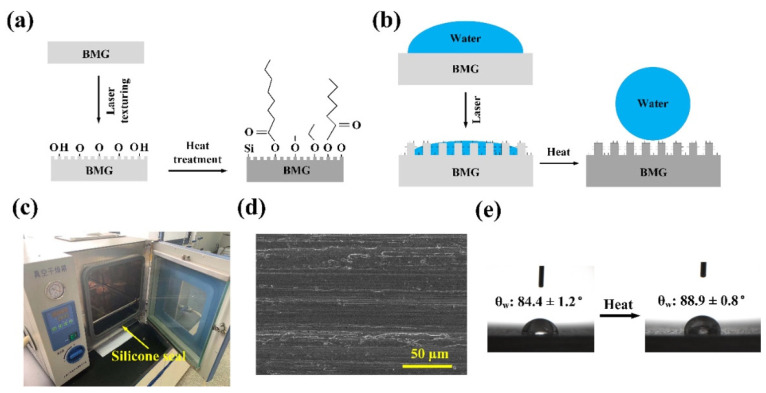
(**a**,**b**) Alteration of surface chemistry and surface wettability; (**c**) the furnace used for heat treatment; (**d**) untreated BMG surface; (**e**) contact angle measurement for the untreated and heat-treated BMG surface [126]. (Copyright: MDPI, Open Access).

Wang et al. (2021) [126] applied a facile laser for functional surface texturing on bulk metallic glass with the goal of modifying surface hardness and surface wettability. It was reported that immediately after machining using the laser, the machined surface became super hydrophilic. However, the surface wettability could be changed using heat treatment, as the laser-textured hydrophilic surface turned into a hydrophobic surface after heat treatment. It was claimed that the laser-induced surface texture, along with surface chemistry and increased surface microhardness, had shown the prospect of using the laser for functional surface modification of BMG. Figure 33 shows the mechanism of wettability transition from hydrophilic to hydrophobic using the combined action of laser surface irradiation and heat transfer [126].

**Figure 34:**
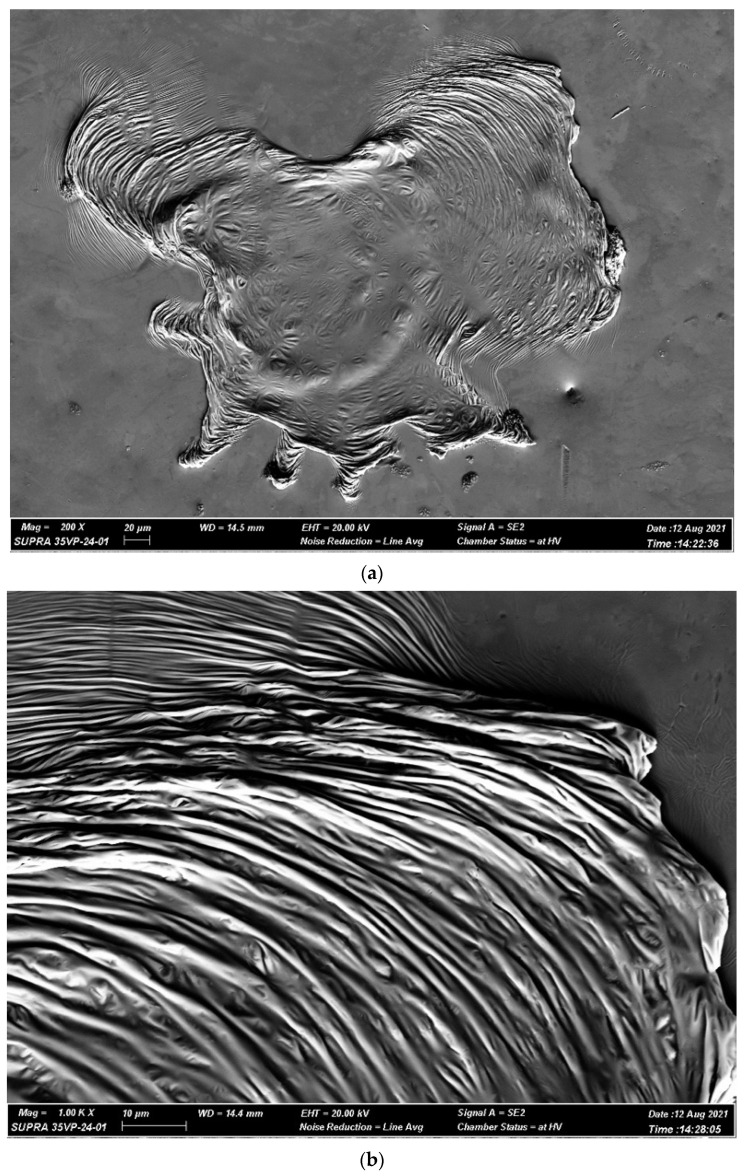
SEM image of (**a**) a crater formed and (**b**) ripple formation at the crater boundary using the laser energy of 0.053 J and lens-to-sample distance of 150 mm (focal length of the lens) [127]. (Copyright: Authors, ASME).

Ma et al. (2022) [127] investigated surface modification of “Vitreloy 1b” BMG (Zr_67_Cu_l0.6_Ni_9_._8_Ti_8.8_Be_3.8_) using a nanosecond laser with 1064 nm wavelength. The effects of laser parameters, such as laser power energy level, focal length on the crater, and slot formation, were studied. One important observation during laser machining of BMG was found to be the ripple formation irrespective of the laser parameter settings. The ripple formation was found to be mostly around the rim of the craters, formed due to laser ablation, and the intensity of ripples was found to depend on laser parameters. The crater depth was found to be the highest for the lens-to-sample distance, equaling the focal length, and the crater depth reduced when the lens-to-sample distance became higher than the focal length of the laser. The diameter of the individual crater increased with the increase of laser energy level and lens-to-sample distance. It was also reported that the relative percentage of Zr increased on the laser-modified surface compared to the unmachined surface of BMG. Figure 34 shows a sample crater formed on the BMG surface using a laser energy of 0.053 J and lens-to-sample distance of 150 mm, which is also the focal length of the lens [127]. The crater shows clear ripple formation at the edge of the crater, while providing very smooth surface in the middle.

Melkani et al. (2022) [128] used a Yb-fiber laser and electro-jet milling (EJM) processes to machine grooves on the Zr-based BMG. It was reported that EJM process reduced or minimized the dross at the groove edges, which was comparatively more visible for laser machining of BMG. It was also reported that the surface microhardness increased due to shear band formation during machining of BMGs.

Song et al. (2022) [129] investigated the machinability response of amorphous and crystalline Zr-based bulk metallic glass under nanosecond laser pulses. It was found that amorphous BMG produced comparatively smaller crater sizes and a smaller heat-affected zone compared to their crystalline counterpart. This may be due to a comparatively higher degree of superheating during laser machining of amorphous BMG. It was also reported that the laser ablation process demonstrated the capability of producing both hydrophilic and hydrophobic surfaces by adjusting the structures produced on the BMG surfaces.

Li et al. (2021) [130] used femtosecond laser double-pulse irradiation to polish surfaces of bulk metallic glass without inducing crystallization on the sub-surface, which is common when polishing BMG with mechanical polishing methods. It was reported that nanoscale surface finish can be achieved using the proposed technique with an optimized setting of laser energy of 0.06 μJ, energy ratio of 40%, and pulse time delay of 20 ps. It was reported that the crystallization of BMG can be reduced at the polished surface and sub-surface due to rapid heating and cooling generated by the femtosecond double-pulse laser irradiation.

### 4.5. Abrasive Waterjet Machining of Bulk Metallic Glasses

In abrasive water jet cutting, water along with abrasive particles is impacted on the surface at a high working pressure to remove materials. An abrasive particle size of less than 5 microns in diameter mixed with water at a working pressure of 1–10 Kbar usually flows through a small-diameter nozzle (of about 250–500 microns) to create a jet stream that can be useful for cutting various materials, such as wood, plastics, metal, and ceramics. Abrasive water jet machining was found to be suitable for machining hard and difficult-to-cut materials. Therefore, researchers have tried machining BMG using abrasive water jet machining.

Wessels et al. (2012) [131] investigated the machinability of Zr_52.5_Cu_17.9_Ni_14.6_Al_10_Ti_5_ BMG using various machining processes, such as dry milling, wire EDM, nanosecond pulse laser micromachining, and AWJ machining. As most of the machining processes cause crystallization of the amorphous structure of BMG at the machined surface or sub-surface, abrasive water jet machining was investigated by the authors. In AWJ processes, garnet micro particles were used as abrasives and the parameters used were as follows: flow rate of 170 g/min, 2400 bar pressure, nozzle diameter of 0.5 mm, and feed rate range 300–1000 mm/min. During machining, thermal imaging was performed using an infra-red camera, which showed a temperature rise of 63.1 degrees during machining. This indicates the possibility of using AWJ machining for BMG without any thermal damage or crystallization. In addition, XRD analysis suggests that BMG does not experience crystallization during AWJ machining due to the absence of Bragg peaks. On the other hand, wire EDM as well as dry milling processes at lower cutting speeds showed the presence of Bragg peaks, indicating the crystallization of bulk metallic glass. With AWJ, fabrication of an orthopedic screw was demonstrated with a low process temperature and without any crystallization.

Loc and Shiou (2012) [132] exploited abrasive water jet polishing on Zr-based BMG materials. Using Taguchi’s L18 design, polishing parameters such as hydraulic pressure, impact angle, standoff distance, abrasive materials, concentration, and polishing time were optimized. Their study demonstrated an improvement in surface roughness from Ra = 0.675 µm to Ra = 0.016 µm for Zr-based BMG after abrasive water jet polishing and reported the removal of the pre-grinded layer after machining. Polishing time, abrasive type, and standoff distance appeared to be the factors significantly affecting the surface roughness of BMG after AWJ polishing.

In another study, the same group of researchers led by Shiou et al. [133] proposed improvement of surface finish of BMG using sequential abrasive jet polishing and an annealing process. It was reported that the surface roughness was reduced from 0.675 to 0.016 μm using abrasive water jet polishing, which could be further lowered to 2 nm using optimal heat treatment parameters. Table 2 presents a summary of the review works on non-conventional machining of BMGs and how it can resolve some of the challenges reported in conventional machining of BMGs.

## 5. Conclusions, Analysis of Current Research State, and Future Directions

Bulk metallic glasses (BMGs) are relatively new materials and have not received much attention in the field of engineering. BMGs possess excellent mechanical properties and have many current and future applications in the field of aerospace and biomedical industries. However, not many research works exist to tackle the machining issues that come with the excellent mechanical properties of BMGs, especially the amorphous structure of BMG that is responsible for the majority of its important properties. While metallic glasses have gained distinction over the years, there are still many challenges associated with machinability of metallic glasses, and the following points provide researchers with directions for some of the unresolved work in the field of the machining of metallic glasses.

**Figure 35:**
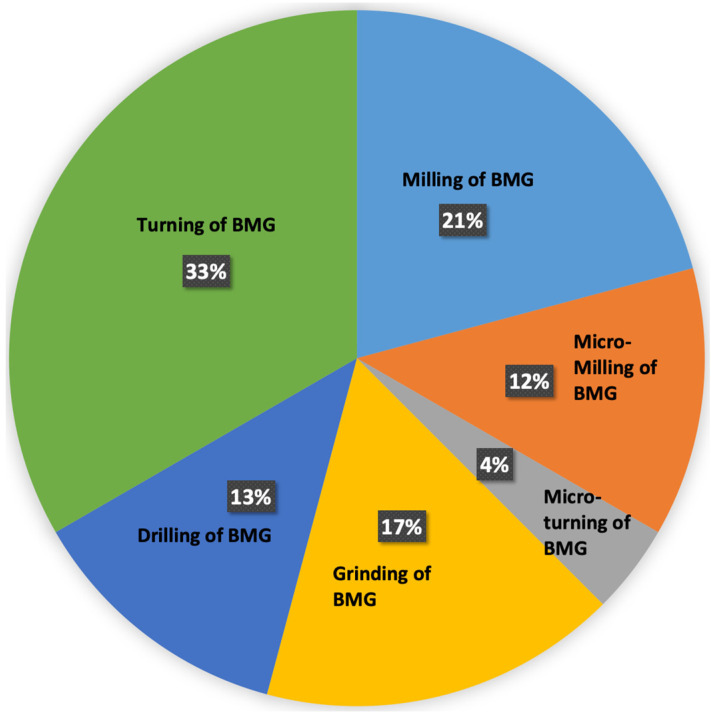
Relative proportion of research studies carried out in different areas of conventional machining of BMG.

**Figure 36:**
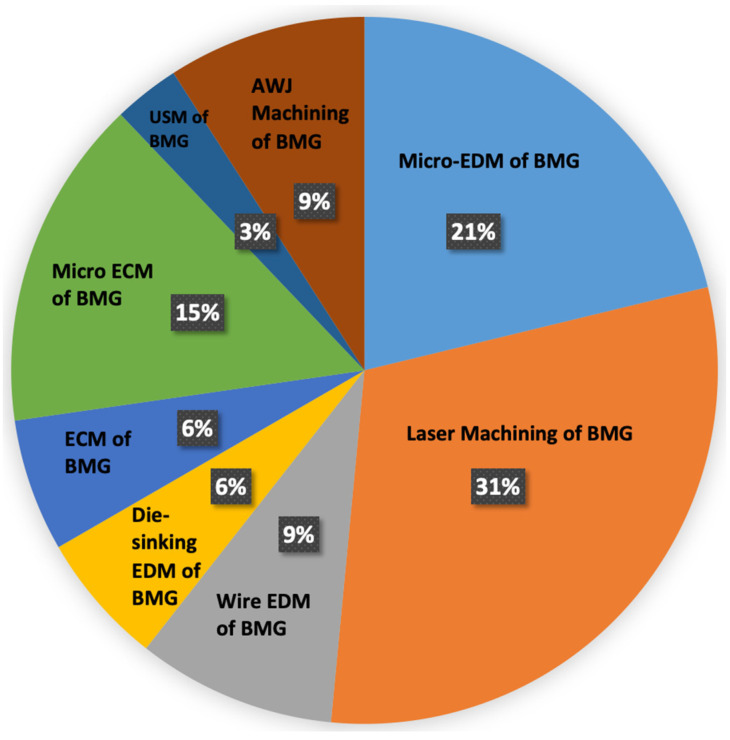
Relative proportion of research studies carried out in different areas of non-conventional machining of BMG.

**Figure 37:**
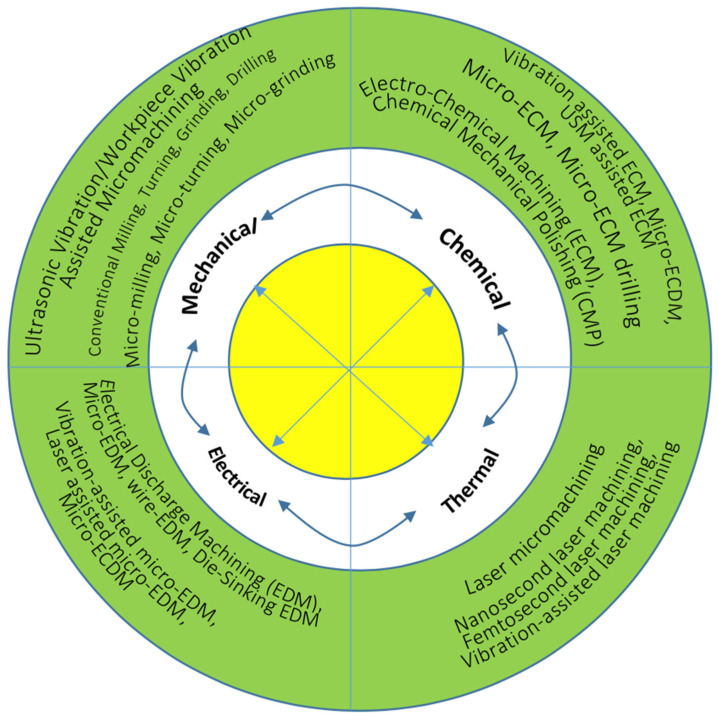
Future research directions proposing various multi-process and hybrid machining processes for successful machining of BMG.

- From the literature, it was found that non-conventional micromachining processes are currently preferable over conventional micromachining processes, mostly because of the capability of non-conventional processes to machine BMGs regardless of their hardness and strengths. The traditional machining of BMGs is also associated with high cost, waste of materials, and oxidation and crystallization of BMGs. Some of the common challenges associated with traditional machining of BMGs are lightning chips causing fire hazards, extensive tool wear and breakage resulting in increase in production cost, and poor surface finish requiring post-process finishing of the parts. The most important challenge is the crystallization of sub-surface microstructure, which changes the unique properties of BMGs. As can be seen from Figure 35 and Figure 36, a significant number of research studies have been carried out on the machining of BMGs using various non-traditional manufacturing processes. Among the non-traditional processes, laser, EDM, and ECM processes were found to be popular in machining of BMGs, mostly because of the non-contact nature of their material removal. However, the non-traditional machining processes, although they can machine BMGs effectively, are not immune when it comes to the crystallization of the machined surface and sub-surface after machining. Therefore, future research should concentrate foremost on eliminating or at least minimizing the crystallization layer on the surface and sub-surface of BMGs after machining.
- Micro EDM processes provided, comparatively, a better alternative to conventional machining than most of the other non-traditional machining processes. Micro EDM is capable of minimizing the discharge energy to a great extent, which allows for the generation of 1–2 μm crater sizes and fewer than 5 microns of recast layer, thus minimizing the crystallization at the subsurface of BMGs. Additionally, micro EDM is capable of producing products with high aspect ratios, low cost, and better surface morphology. Due to the capability of micro EDM to machine BMGs without affecting the crystallization significantly, it can be used to machine features in BMGs with a fine surface finish while keeping the mechanical properties of BMGs unaffected. Future research should focus on applying the assistance of external sources to further improve the surface finish and minimize crystallization. Two of the most effective hybrid micromachining processes that were reported in literature are vibration-assisted micro EDM [134] and powder mixed micro EDM [135]. In addition, other micro-EDM-based hybrid machining processes, as discussed in a recent publication by the authors [136], can be applied to further improve the micro EDM machinability of BMGs. Figure 36 proposed some EDM-based hybrid machining processes that can be applied to further improve the machining performance of electrical and thermal machining processes for successfully machining BMGs.
- Very few studies have been conducted using conventional processes, especially at the micro scale, as can be seen from Figure 34. It can be seen that there have been a few studies on micro-milling of BMGs, with almost no attempt to machine BMGs with other micro-scale mechanical machining processes. This is mainly due to the very high cutting forces, lightning chip generation, and light emissions during the drilling of BMGs. In addition, very few studies have focused on the drilling of BMGs using mechanical drilling processes due to significant challenges. As a consequence of this finding, researchers or industry professionals can consider pre-hole drilling using the micro EDM process before performing the actual mechanical drilling process with target dimensions when drilling holes in BMGs. Therefore, future research studies should converge on traditional drilling with non-traditional drilling processes (such as micro EDM drilling, laser drilling, USM, etc.) to complement each other’s shortcomings/challenges and, thus, to improve overall machining performance. In addition, the micro scale machinability of BMGs needs to be explored in more depth in future research studies. Figure 36 proposes how the machining performance of mechanical-contact-based machining processes can be improved by incorporating other sources of energy, such as chemical, electrical, thermal, etc., to innovate new machining processes for BMGs.
- Machining of BMGs is expensive due not only to the cost associated with machining but also due to the cost of BMG itself. One way to reduce costs is to minimize the number of experiments and carry out modeling and simulation studies on the machining of BMGs. Currently, very few research studies have focused on modeling and simulation of machining of BMGs. Future research should focus on using computer-aided design (CAD) and computer-aided machining (CAM) to design optimum machining conditions for machining BMGs and conducting simulations using advanced simulation techniques such as finite element analysis (FEA) or molecular dynamics simulations (MDS) to narrow down experimental procedures and lower costs. Apart from the initial cost of modeling and simulation software, the cost of BMG materials, cutting tools, machine run time, operator costs, and other maintenance costs can be reduced, thus reducing the overall production cost for product development using BMGs.
- Most of the research studies on micro ECM of BMGs showed that corrosion and transpassive dissolution processes were major challenges during micro ECM of BMGs. Therefore, more research studies should be conducted on developing innovative strategies to minimize corrosion and the transpassive dissolution mechanism. Some of the suggestions are as follows: selection of electrolytes that do not promote these two phenomena and removal of the corrosive layer from the machined surface by micro-ECM-based hybrid processes. Some hybrid processes that could potentially be combined with micro ECM are combined micro ECM and micro-grinding, abrasive micro-jet electro-chemical machining, abrasive mixed electro-chemical grinding, etc. All of these processes involve secondary material removal and work as finishing processes, which will be effective in the removal of the corrosive layer from the BMGs. Figure 37 proposes some innovative hybrid machining processes that can be explored to improve the machinability of BMGs in chemical machining processes. The unwanted corrosion and transpassive dissolution during micro ECM of BMGs poses a major challenge to obtaining a high level of dimensional accuracy and also induces an oxide layer on the machined surfaces of BMGs. A hybrid process combining a post-processing finishing process along with micro ECM (mechanical finishing processes like grinding, ultrasonic machining, abrasive finishing, etc.) will allow removal of oxide and corroded layers and, thus, will enhance overall surface finish and dimensional accuracy of the machined BMG parts.
- It is obvious now that by reducing cutting forces for conventional machining and discharge energy level for EDM, the degree of crystallization can be reduced. So far, no or very few research studies have focused on nanomachining of BMGs. Nano-EDM uses energy in nano Joules (nJ), which can significantly reduce the crystallization on the machined surface and sub-surface. Nano EDM was found to successfully machine electrically conductive metals and is capable of producing sub 100 nm features with consistency and repeatability [137]. In addition, nano scale conventional machining, such as tip-based nanomachining, can be introduced for machining BMGs with minimal to no crystallization.
- One of the potential areas of application of BMGs that is seeing growth is using BMG implants for biomedical applications. BMGs have all of the important properties a biomedical implant need to have, such as high strength, hardness, toughness, corrosion resistance, etc. In recent years, there have been several research studies focusing on investigating the biocompatibility of BMGs after being machined by both conventional and non-conventional machining processes. One of the studies from the authors has reported application of micro EDM for surface generation with improved biomedical characteristics [138]. Future research should investigate enhancing the biomedical characteristics of BMGs using innovative manufacturing processes. BMGs have superior strength and toughness compared to the two most common implant materials: titanium alloy and steel. However, they are susceptible to brittle failure, which can be an issue when using BMGs as biomedical implants. The research on manufacturability and performance of BMGs as biomedical implants will have significant implications for the growth of the biomedical implant industry.
- Monitoring, control, and feedback to improve machining performance has remained one of the major and important research areas in the manufacturing field. Although there have been many studies on improving machining performance and optimizing process parameters using in situ monitoring and control, very few studies focused on applying those techniques for conventional or non-conventional machining of BMGs. In addition, application of artificial intelligence (AI) in machining has been one of the very recent developments in the field of manufacturing, which needs extensive research work [139]. Therefore, future researchers should focus on applying machine learning (ML), artificial intelligence (AI), and digital twins to the machining of BMGs to optimize the machining performance and reduce the crystallization upon machining, thus protecting the excellent mechanical properties of BMCs for prospective applications of machined parts.
- The laser bed fusion (LBF) technique, which is one of the very popular additive manufacturing technologies, can produce metallic, polymer, and ceramic components by selectively melting a powder of the concerned materials on a build platform (heated/unheated) with the help of a scanning laser beam layer by layer as per the design of a three-dimensional CAD file [140,141]. The extreme fast cooling constraint offered by the conventional BMG fabrication technique during large BMG fabrication can be overcome by laser powder bed fusion of BMG, where a part bigger than the critical casted size can be produced from a combination of different elemental powders. Moreover, due to its additive nature, laser bed fusion techniques offer the advantage of producing geometrically complex parts as well as a wide range of BMGs, either with high or low glass forming ability [142]. In addition, preproduction and post-production tooling may not be necessary, therefore making this technology more cost effective [143]. Additively manufactured BMGs hold huge potential, not only for patient-specific application in the biomedical field [144], but also for applications in aerospace [145] and jewelry industries [146]. However, LBF still deals with issues such as partial crystallization, degrading bio response behavior, undesired pores, and microcracks [147]. As a result, future research should focus on design for additive manufacturing, investigating the feasibility of replacing existing BMG products using the existing metal additive manufacturing processes, or innovating novel AM processes for printing industrial grade BMG parts.

## Figures and Tables

**Table 1:** Summary of the research findings on conventional machining of BMG.

Process	BMG	Type of Machining	Remarks: Challenges Faced and Measures Taken to Resolve
Milling	Zr-based BMG	Micro-milling	Constant spindle speed with high feed rate changed the structure of BMG from amorphous to crystalline. High feed rate and spindle speed produced light with extensive intensity due to heat accumulation [4].
		FEM simulation slot milling	Low feed rate simulation could not provide comprehensive results due to lack of various frictions in the simulation and roundness of tool with smaller chip thickness [67]. Lower feed rate resulted in reduction of chip thickness.
		Ion milling	Specimen produced using ion milling had substantial crystallization and highest nominal temperature induction. Use of nitrogen for ion milling showed non-uniform local heating [68].
		Milling holes	Stress distribution resulting from the artificial holes could be responsible for enhanced mechanical characteristics. The specimens with artificial holes had plasticity increased by 2–4.5% without compromising the fracture toughness of the material [69].
Turning	Zr-based BMG	Turning	The test revealed that high-temperature machining leads to oxidation of crystallization. Low cutting speed showed no crystallization on both the machined surface and the chips that were produced. The heat accumulation in high cutting speed led to oxidation of the chips [5].
			High cutting speed and low thermal conductivity were found to trigger light emission and oxide formation on chips. Cutting force was seen to be reduced due to thermal softening at high speed. Chip formation was highly affected by maximum shear stress because of BMG’s amorphous structure [75].
			The low roughness was achieved with high feed rates and spindle speed using single crystal diamond tool. Large depth of cut resulted in sinusoidal grooves, while high feed rate resulted in irregular grooves on the surface [77].
	ZrTiAlCuNi metallic glass	Turning	Production of lamellar chip was due to thermal instability resulting in shear localization, which is explained by bifurcation phenomenon. High free volume concentration with high Poisson ratio resulted in reduction of continuous lamellar chip formation [74].
Drilling	Zn-based BMG		Long tangled ribbon chips were formed with higher spindle speed while long spiral chips were formed with slow feed rates. High feed rates seemed to reduce the size of burr in both entry and exit areas [3].
			Significant tool wear is seen with high speed, while low speed resulted in built up edges in the work material and damaged the drill bits because of extremity in cutting force [84].
Grinding	Zr_52.5_Ti_5_Cu_17.9_Ni_14.6_Al_10_		Higher feed rate also comes with larger chip thickness, which in turn causes reduced ploughing action. Consequently, the specific energy for ploughing as well as total specific energy falls. Ductile chip formation and ploughing mechanism are the main causes behind the material removal of BMG [86].
			Grinding force shows decreasing trend as the specific grinding energy increases along with the increase in cutting speed. Reduced surface roughness is observed with increased cutting speed [87].
	(BMG^C^ Zr_70_Ni_10_Cu_20_)_82_Ta_8_Al_10_ and BMG^D^ Zr_55_Cu_30_Ni_5_Al_10_).		Increased spindle speed causes increase in total abrasive number per unit time, which eventually reduces grinding force. Increased total abrasive number results in finely divided grinding chips as well as increased abrasive involvement in sliding and ploughing. Increased feed rate increases the micro-grinding force as well as roughness due to the increase in arithmetic deviation of surface contour, which occurs due to the increase in uncut chip thickness and reduced contact point between abrasive surfaces of grinding tool and workpiece [88].

**Table 2:** Summary of the research findings on non-conventional machining of BMGs.

Process	BMG	Type of Operation	Remarks: Challenges Faced and Measures Taken to Resolve
EDM	Zr-based BMG	Micro EDM	MRR increases with capacitance and voltage due to increased discharge energy. Decreased voltage results in tapered shape due to increased wear and decreased roughness due to reduced crater size. Micro EDM retains the amorphous structure of BMG more than conventional EDM [100].
		Micro EDM grinding	Carbon-free electrodes caused crystallization. Decrease in voltage causes increase in surface roughness; decrease in capacitance has opposite trend. Micro EDM grinding can be an effective tool to remove carbonized and crystallized surfaces [101].
	La_62_Al_14_Ni_12_Cu_12_,Zr_55_Al_10_Ni_5_Cu_30_,Cu_46_Zr_44_Al_7_Y_3_	Micro EDM	Highest heat-affected zone was observed for BMG with low thermal conductivity. All three alloys showed no devitrification [102].
	Pd-Cu-P-Ni		Increase in depth results in shallow crates, with increased carbon deposition. Cu and P phase were formed and crystallization was observed in the machining process [103].
ECM	Zr-based BMG		NaNO_3_ was not a suitable electrolyte for machining due to the formation of corrosive layers on the surface of the machined parts. Controlling fast transpassive dissolution process, optimizing electrode distance, use of electrolytes, and pulse parameters enhanced the machining process [113].
			Micro tool electrode caused little to no crystallization. Re-passivation was prevented using voltage of 2.235 V. The use of 1:10 duty cycle and 0.4 L/min flow rate produced a shape with good tolerance and finish [117].
	Ni-based metallic glass		Tool wear and heat induction were not observed and the material removal rate was not affected by the hardness of the metal. NaNO_3_ produced a lower aspect ratio compared to sulfuric acid. Surface finish was found to be desirable in electrolytes of high concentration. Efficiency and stability of machining aws improved with voltage pulse rise time of 2 ns [114].
			Surface was found to be smoother when sulfuric acid in 0.1 M concentration was used, HCl at 0.1 M surface roughness was found to be higher. High current density enhanced surface morphology as the voltage and feed rate were larger with high current [118].
			Mass transport with the use of CNF was improved as the electrolyte was destabilized quickly and the irregular motion of electrolysis was also accelerated. Stable passive film was formed with use of sulfuric acid, which in turn increased surface finish and localization. CNF was found to be a great electrode in machining of nickel-based metallic glass [115].
			Increase in feed rate reduced the machined gap. Short pulse duration with low feed rate helped in producing smaller side gap. With pulse duration held constant, increasing pulse period provided better accuracy. Low concentration of electrolyte produced better slit width [116].
	Fe-based BMG		Low pulse-off voltages resulted in no material removal. High pulse-on and pulse-off voltages resulted in irregular structures. Sulphuric acid with an addition of Fe_3_(SO_4_)_3_ reduced evolution of hydrogen [119].
Ultrasonic machining	ZrCuTi metallic glass		It was seen that overcut was decreasing with increase in feed rate. Lower slurry rate and higher feed rate tended to increase material removal rate. Tapered angle was found to be higher with higher grits [120].
Laser machining	Mg-Cu-Gd	355 nm ultraviolet laser and 1064 nm infrared laser	For UV laser, it was found that increase in scan speed and laser power decreased the desired cutting depth. Amorphous structure turned into crystalline structure when the laser fluence was increased and the scanning speed was increased. Micromachining with UV produced optimal result [123].
	Zr-based bulk metallic glass	Nanosecond laser	Nanosecond laser preserved the mechanical properties of Zr BMG. Thermal diffusion reduction led to better morphology, and this can be obtained by using low peak power, low pulse frequency, and higher track distance [121].
Abrasive waterjet machining	Zr_52.5_Cu_17.9_Ni_14.6_Al_10_Ti_5_BMG	Dry milling, wire EDM, nanosecond pulsed laser micromachining, and AWJ	XRD analysis suggests that BMG does not experience crystallization during AWJ machining due to the absence of Bragg peaks. Fabrication of orthopedic screw was demonstrated with low process temperature and without any crystallization [131].
	Zr-based BMG	Taguchi’s L18 design	Polishing time, abrasive type, and standoff distance appeared to be the factors significantly affecting the roughness [132].

## Data Availability

Data available on request.
